# Mature dendritic cells correlate with favorable immune infiltrate and improved prognosis in ovarian carcinoma patients

**DOI:** 10.1186/s40425-018-0446-3

**Published:** 2018-12-04

**Authors:** Iva Truxova, Lenka Kasikova, Michal Hensler, Petr Skapa, Jan Laco, Ladislav Pecen, Lucie Belicova, Ivan Praznovec, Michael J. Halaska, Tomas Brtnicky, Eva Salkova, Lukas Rob, Roman Kodet, Jeremy Goc, Catherine Sautes-Fridman, Wolf Herman Fridman, Ales Ryska, Lorenzo Galluzzi, Radek Spisek, Jitka Fucikova

**Affiliations:** 1 Department of Immunology, Charles University, 2nd Faculty of Medicine and University Hospital Motol, Prague, Czech Republic; 2 Sotio, Prague, Czech Republic; 3 Department of Pathology and Molecular Medicine, Charles University, 2nd Faculty of Medicine and University Hospital Motol, Prague, Czech Republic; 4 The Fingerland Department of Pathology, Charles University, Faculty of Medicine and University Hospital Hradec Kralove, Hradec Kralove, Czech Republic; 5 Department of Gynecology and Obstetrics, Charles University, Faculty of Medicine and University Hospital Hradec Kralove, Hradec Kralove, Czech Republic; 6 Department of Gynecology and Obstetrics, Charles University, 3rd Faculty of Medicine and University Hospital Kralovske Vinohrady, Prague, Czech Republic; 7 Department of Gynecology and Obstetrics, Charles University, 2nd Faculty of Medicine and University Hospital Motol, Prague, Czech Republic; 8 INSERM, U1138, Centre de Recherche des Cordeliers, Paris, France; 9 Sorbonne Université, Paris, France; 10 Université Paris Descartes/Paris V, Paris, France; 11 Department of Radiation Oncology, Weill Cornell Medical College, New York, NY USA; 12 Sandra and Edward Meyer Cancer Center, New York, NY USA

**Keywords:** Dendritic cells, DC-LAMP, CD8^+^ cytotoxic T lymphocytes, Natural killer cells, Tertiary lymphoid structures

## Abstract

A high density of tumor-infiltrating CD8^+^ T cells and CD20^+^ B cells correlates with prolonged survival in patients with a wide variety of human cancers, including high-grade serous ovarian carcinoma (HGSC). However, the potential impact of mature dendritic cells (DCs) in shaping the immune contexture of HGSC, their role in the establishment of T cell-dependent antitumor immunity, and their potential prognostic value for HGSC patients remain unclear. We harnessed immunohistochemical tests and biomolecular analyses to demonstrate that a high density of tumor-infiltrating DC-LAMP^+^ DCs is robustly associated with an immune contexture characterized by T_H_1 polarization and cytotoxic activity. We showed that both mature DCs and CD20^+^ B cells play a critical role in the generation of a clinically-favorable cytotoxic immune response in HGSC microenvironment. In line with this notion, robust tumor infiltration by both DC-LAMP^+^ DCs and CD20^+^ B cells was associated with most favorable overall survival in two independent cohorts of chemotherapy-naïve HGSC patients. Our findings suggest that the presence of mature, DC-LAMP^+^ DCs in the tumor microenvironment may represent a novel, powerful prognostic biomarker for HGSC patients that reflects the activation of clinically-relevant anticancer immunity.

**Electronic supplementary material:**

The online version of this article (10.1186/s40425-018-0446-3) contains supplementary material, which is available to authorized users.

## Introduction

Tumors emerge and evolve in the context of a complex metabolic, trophic and immunological crosstalk with cells of different types, including (but not limited to) epithelial, endothelial, stromal and immune cells [1, 2]. Thus, the microenvironment of solid malignancies exhibit a considerable degree of heterogeneity, not only across different types of disease, but also across the same tumor type in different patients or even different malignant lesions in the same individual [3]. Not surprisingly, both non-immunological and immunological components of the tumor microenvironment (TME) have been attributed robust prognostic and/or predictive value in multiple cohorts of patients with cancer [4–8]. In particular, high levels of tumor-infiltrating CD8^+^ T lymphocytes (CTLs), which are key mediators of anticancer immunity, are strongly associated with prolonged survival in patients affected by various solid tumors including high-grade serous ovarian carcinoma (HGSC) [9–12]. Intriguingly, the majority of HGSCs containing high frequencies of CD8^+^ CTLs are also robustly infiltrated by CD20^+^ B cells [13], and patients whose tumors exhibit such an abundant co-infiltration have higher survival rates than patients with tumors that only contain high amounts of CD8^+^ CTLs [14, 15]. That said, the cellular mechanisms that govern the recruitment of CD8^+^ CTLs and CD20^+^ B cells to the microenvironment of HGSCs and their activation remain unclear.

It has previously been shown that CD8^+^ and CD20^+^ cells often co-localize in lymphoid aggregates of different sizes and morphologies in the HGSC microenvironment [15]. These aggregates, which have previously been identified as tertiary lymphoid structures (TLSs), are known for their ability to initiate tumor-targeting immunity and for their positive prognostic value in patients with various tumor types [16–19]. TLSs harbor indeed prominent B-cell follicles adjacent to discrete zones containing CD4^+^ and CD8^+^ T cells, dendritic cells (DCs) and high endothelial venules [18, 20]. Since mature DCs selectively home to TLS, they constitute a reliable and specific marker of these structures [21, 22]. Nevertheless, the impact of mature DCs on the composition and functional orientation of the tumor infiltrate, their ability to drive T cell-dependent anticancer immunity, and their potential prognostic and predictive value in the setting of HGSC remain to be deciphered.

Here, we investigated the clinical impact of tumor infiltration by mature DCs in three independent cohorts of 81, 66 and 20 patients with resectable HGSC who did not received neoadjuvant chemotherapy. Our data suggest that while both mature DC-LAMP^+^ DCs and CD20^+^ B cells participate in the generation of anticancer immunity, only the former are critical for licensing a CTL-dependent tumor-targeting immune response that translates into clinical benefit for HGSC patients.

## Materials and methods

### Patients

***Study groups 1 and 2.*** Two retrospective series of 81 and 66 formalin-fixed paraffin-embedded (PPFE) samples were obtained from patients with HGSC who underwent primary surgery in the absence of neoadjuvant chemotherapy between 2008 and 2014 at University Hospital Hradec Kralove and University Hospital Motol (Czech Republic). Baseline characteristics of these patients are summarized in Table 1 and Additional file 1**:** Table S1, respectively. Pathologic staging was performed according to the 8th TNM classification (2017), and histologic types were determined according to the current WHO classification [23, 24]. Data on long-term clinical outcome were obtained retrospectively by interrogation of municipality registers or the patient’s families. ***Study group 3.*** An additional series of 20 samples from HGSC patients was prospectively collected at Hospital Motol (Czech Republic) (Additional file 1**:** Table S5). ***Study group for NGS.*** 18 HGSC patient samples collected at Hospital Motol (Czech Republic) were used for NGS data analysis (Additional file 1**:** Table S6). Written informed consent was obtained from patients before inclusion in the study. The protocol was approved by the local ethics committee.

**Table 1:** Main clinical and biological characteristics of 81 HGSC patients enrolled in the study (University Hospital Hradec Kralove)

Variable	Overall cohort (*n* = 81)
Age	
Mean age (y) ± SEM	61 ± 10
Range	31–84
pTNM stage	
Stage I	20 (24,5%)
Stage II	6 (7,4%)
Stage III and IV	55 (68,1%)
Debulking	
R0	38 (47,0%)
R1	11 (13,5%)
R2	32 (39,5%)
Vital status of patients	39 (47,5%)

### Immunohistochemistry

Tumor specimens from study groups 1 and 2 were fixed in neutral buffered 10% formalin solution and embedded in paraffin as per standard procedures. Immunostaining with antibodies specific for lysosomal associated membrane protein 3 (LAMP3; best known as DC-LAMP), CD8, CD20 and natural cytotoxicity triggering receptor 1 (NCR1; best known as NKp46) (Additional file 1**:** Table S2) was performed according to conventional protocols. Briefly, tissue sections were deparaffinized, followed by antigen retrieval with Target Retrieval Solution (Leica) in EDTA pH 8 in preheated water bath (98 °C, 30 min). The sections were allowed to cool down to RT for 30 min, and endogenous peroxidase was blocked with 3% H_2_O_2_ for 15 min. Thereafter, sections were treated with protein block (DAKO) for 15 min and incubated with primary antibodies, followed by the revelation of enzymatic activity. Images were acquired using a Leica Aperio AT2 scanner (Leica).

### Cell quantification

DC-LAMP^+^ cells were differentially quantified in the tumor stroma and tumor nests of the whole section with Calopix (Tribvn). The total number of CD8, NKp46 and CD20 density was analyzed on the entire tumor section of all 81 HGSC patients using Calopix software without further division for tumor stroma and tumor nest based on previous published protocols [14, 22, 25]. The NKp46 staining was performed based on previously optimized and published protocols [22, 25] and appropriate isotype controls were used throughout the study. The antibody against NKp46/NCR1 (R&D), clone 195,314 is suitable for immunohistochemistry analysis as indicated by manufacturer. Data are reported as absolute number of positive cells/mm^2^ (for DC-LAMP^+^, CD8^+^ NKp46^+^ cells) or cell surface/total tumor section surface (for CD20^+^ cells), as previously described [14, 21]. TLS were enumerated in whole sections using upon assessment of co-localizing DC-LAMP^+^ and CD20^+^ B cells, as previously described [14, 21]. Immunostaining and quantifications were reviewed by at least three independent observers (IT, LK, JF, PS, JL) and an expert pathologist (JL, PS).

### Flow cytometry

Total live mononuclear cells were isolated from fresh tumor specimens, as previously described [26]. Mononuclear cells were stained with several panels of fluorescent primary antibodies (Additional file 1**:** Table S3) or appropriate isotype controls for 20 min at 4 °C in the dark, following by washing and acquisition on a Fortessa flow cytometer (BD Bioscience). Flow cytometry data were analyzed with the FlowJo software (TreeStar). Gating strategies are depicted in Additional file 1**:** Figure S3A and 3C.

### Degranulation and IFN-γ production after in vitro stimulation

Mononuclear cells isolated from fresh tumor specimens were stimulated with 50 ng/mL phorbol 12-myristate 13-acetate (PMA) + 1 μg/ml ionomycin in the presence of anti-CD107a FITC monoclonal antibody (BioLegend) for 1 h followed by 3 h incubation with brefeldin A (BioLegend). Unstimulated cells were used as control. Cells were then washed in PBS, stained with anti-CD45 PerCP (EXBIO), anti-CD3 Alexa Fluor 700 (EXBIO) or APC-eFluor780 (eBioscience), anti-CD4 ECD (Beckman Coulter), anti-CD8 HV500 (BD Biosciences) and anti-CD56 Alexa Fluor 700 (BioLegend) monoclonal antibodies, fixed in fixation/permeabilization buffer (eBioscience), permeabilized with permeabilization buffer (eBioscience) and stained with anti-IFN-γ PE-Cy7 (eBioscience), anti-granzyme B Brilliant Violet 421 (BD Biosciences) and anti-perforin APC (BioLegend) monoclonal antibodies. The percentage of CD3^+^CD8^+^ T cells and CD3^−^CD56^+^ NK cells producing IFN-γ and degranulating upon PMA/ionomycin stimulation were determined by flow cytometry. The data were analyzed with the FlowJo software package (Tree Star, Inc.).

### NGS data analysis

Hierarchical clustering analysis was conducted for differentially expressed genes (DEGs) using the PHEATMAP package in R, based on The Euclidean distance and complete clustering method. GO, KEGG and REACTOME analyses were performed using ClueGo [27]. The MCP-counter R package was used to estimate the abundance of tissue-infiltrating immune cell populations [28].

### Statistical analysis

Survival analysis was performed using the Survival R package, using both log-rank tests and Cox proportional hazard regressions. For log-rank tests, the prognostic value of continuous variables was assessed using median-based cutoffs. For Cox proportional hazard regressions, immune densities were log-transformed. Variables that were not significantly associated with prognosis in univariate Cox regression (Wald test *p* > 0.05), as well as variables that were intrinsically correlated, were not included in multivariate Cox regressions. Fisher’s exact tests, Student’s *t* tests, and the Wilcoxon and Mann-Whitney tests were used to assess statistical significance, *p* values are reported (considered not significant when > 0.05).

## Results

### Prognostic impact of mature DC infiltration in HGSC

**Fig. 1:**
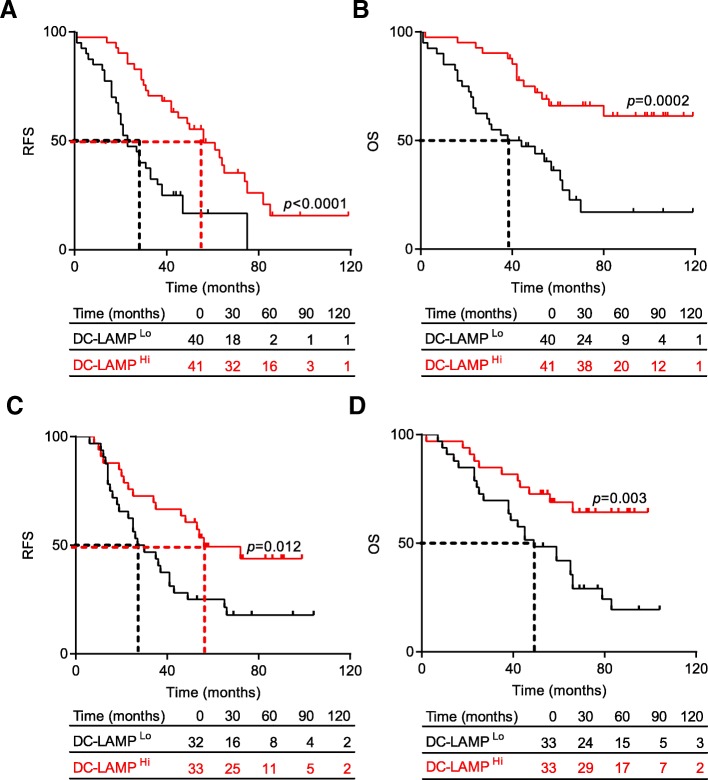
Prognostic impact of mature DCs in the tumor microenvironment of chemotherapy-naive HGSC patients. RFS (**a, c**) and OS (**b, d**) of two cohorts of 81 (**a, b**) and 66 (**c, d**) patients with HGSC who did not receive neoadjuvant chemotherapy, upon stratification based on median density of DC-LAMP^+^ cells in the tumor stroma. Survival curves were estimated by the Kaplan-Meier method, and differences between groups were evaluated using log-rank test. Number of patients at risk are reported

Tumor samples from a retrospective series of 81 patients with HGSC who did not receive neoadjuvant chemotherapy (Table 1), were analyzed for the density of DC-LAMP^+^ DCs by immunohistochemistry (IHC) (Additional file 1: Figure S1A). Although density was relatively heterogeneous across samples, mature DCs were mainly localized to the tumor stroma (median = 3.66 cells/mm^2^) rather than in direct apposition with tumor cell nests (median = 0.42 cells/mm^2^) (Additional file 1**:** Figure S1B). To evaluate the prognostic impact of DC-LAMP^+^ cells in this patient cohort, we stratified it based on median density of DC-LAMP^+^ cells in the tumor stroma and tumor nest, followed by retrospective RFS and OS analysis. Patients with high density of DC-LAMP^+^ cells (DC-LAMP^Hi^) in the tumor stroma exhibited significantly longer RFS and OS as compared to their DC-LAMP^Lo^ counterparts (median RFS: 55 mo. versus 28 mo.; *p* < 0.0001; median OS; > 120 mo. versus 41 mo.; *p* = 0.0002) (Fig. 1 a, b). We also identified a trend toward improved OS (but not RFS) for HGSC patients with a high density of DC-LAMP^+^ DCs in tumor nests (Additional file 1: Figure S1C). Univariate Cox analysis confirmed the prognostic impact of mature DC infiltration in both the tumor stroma (*p* = 0.0008) and the tumor nest (*p* = 0.018) (Table 2). To validate these findings in an independent cohort, we evaluated the prognostic role of mature DCs in a group of 66 patients with HGSC who did not receive neoadjuvant chemotherapy (Additional file 1: Table S1). Also in this set of samples, DC-LAMP^+^ cells were mainly found in the tumor stroma compared to the tumor nest (data not shown). Moreover, low densities of DC-LAMP^+^ cells in the tumor stroma were also associated with an increased risk of relapse and poor prognosis in this cohort (Fig. 1c, d). Indeed, median OS was only 49 mo. for DC-LAMP^Lo^ patients, compared to > 120 mo. for their DC-LAMP^Hi^ counterparts (Fig. 1d). These results demonstrate that the presence of DC-LAMP^+^ cells in the tumor stroma constitutes a strong prognostic biomarker for the identification of HGSC patients with favorable disease outcome upon tumor resection, as confirmed by multivariate Cox analysis (Table 3).

**Table 2:** Univariate Cox proportional hazard analysis

	Overall survival	
Variable	HR (95% Cl)	*p*
Age	1.03 (1.00–1.06)	0.045
CA125	1.23 (0.99–1.54)	0.058
CD8	0.78 (0.63–0.96)	0.022
CD20	0.09 (0.01–0.72)	0.023
DC-LAMP stroma	0.53 (0.37–0.77)	0.0008
DC-LAMP tumor	0.48 (0.27–0.88)	0.018
DC-LAMP summary	0.52 (0.34–0.81)	0.0037
Debulking	1.48 (1.07–2.03)	0.018
NKp46	0.57 (0.25–1.42)	0.24
Stage	2.03 (1.34–3.07)	0.0008

**Table 3:** Multivariate Cox proportional hazard analysis

	Overall survival	
Variable	HR (95% Cl)	*p*
Stage	2.15 (1.31–3.59)	0.0024
DC-LAMP stroma	0.62 (0.44–0.87)	0.0057

To evaluate the density of mature DCs within TLSs as well as the prognostic impact of the latter, we quantified TLSs by examining the co-localization of DC-LAMP^+^ DCs with CD20^+^ B cells. In line with previous findings [15], TLSs were only found in 10% of specimens from both cohorts of HGSC patients included in this study (data not shown). Next, we stratified patients from both cohorts based on the presence of TLSs within their TME and investigated whether TLS^Neg^ and TLS^Pos^ patients differed in terms of OS. The presence of TLS did not influence OS in these retrospective cohorts of HGSC patients (Additional file 1: Figure S2, Table 2).

### Mature DCs correlate with signs of a T_H_1-polarized effector immune response

**Fig. 2:**
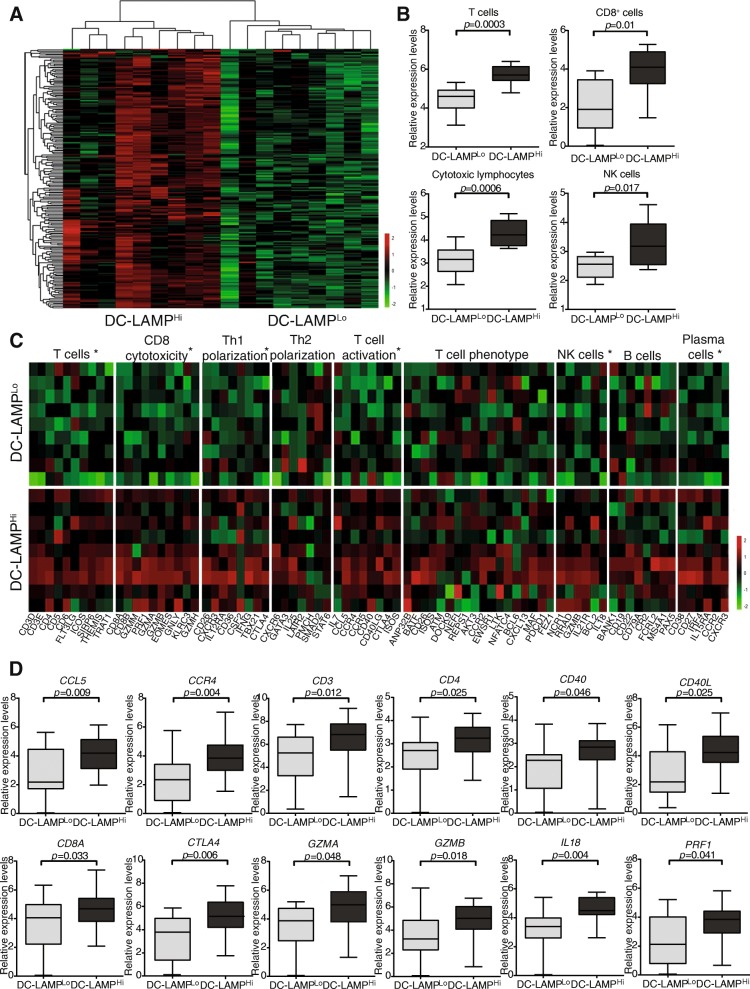
Transcriptional signatures of the tumor microenvironment of DC-LAMP^Hi^ versus DC-LAMP^Lo^ HGSCs. **a** Hierarchical clustering of genes significantly upregulated in 9 DC-LAMP^Hi^ versus 9 DC-LAMP^Lo^ HGSCs, as determined by RNA-Seq. Highly expressed genes are in red, and lowly expressed genes are in green. **b** Relative abundance of T cells, CD8^+^ T cells, cytotoxic lymphocytes and NK cells across DC-LAMP^Hi^ and DC-LAMP^Lo^ HGSCs, as determined by MCP-counter on RNA-Seq data. Box plots: lower quartile, median, upper quartile; whiskers, minimum, maximum. **c** Relative expression levels of genes linked to T cells, CD8 cytotoxicity, T_H_1 polarization, T_H_2 polarization, T cell activation, T cell phenotype, NK cells, B cells and plasma cells in 9 DC-LAMP^Hi^ versus 9 DC-LAMP^Lo^ HGSCs, as determined by RNA-Seq and IHC. Benjamin-Hochberg correction was used for RNA-Seq data. Highly expressed genes are in red, and lowly expressed genes are in green. **d** qRT-PCR-assisted quantification of *CCL5, CCR4, CD3, CD4, CD40, CD40L, CD8A, CTLA4, GZMA, GZMB, IL18* and *PRF1* expression levels in 23 DC-LAMP^Hi^ and 23 DC-LAMP^Lo^ HGSCs patients who did not receive neoadjuvant chemotherapy. Box plots: lower quartile, median, upper quartile; whiskers, minimum, maximum. Ct values were normalized using the global normalization method (*n* = 46 samples). qRT-PCR data were analyzed by unpaired Student’s *t* tests using GenEx software (MultiD Analysis). All values are presented as mean ± SD

To characterize the impact of mature DCs on the composition of the HGSC immune infiltrate, we used RNA-Seq to compare gene expression profiles of 9 DC-LAMP^Lo^ and 9 DC-LAMP^Hi^ patients, as identified by IHC (Additional file 1: Table S6). We identified a set of 199 genes that were significantly overrepresented in from DC-LAMP^Hi^ patients as compared to their DC-LAMP^Lo^ counterparts (Fig. 2a; Additional file 1: Table S4). Functional studies revealed a strong association between DEGs with immune system activation and inflammation. Alongside, we used the MCP-counter R package to estimate the relative abundance of different cell populations in the TME of DC-LAMP^Hi^ versus DC-LAMP^Lo^ patients. Thus, compared to their DC-LAMP^Lo^ counterparts, DC-LAMP^Hi^ tumors exhibited overrepresentation for sets of genes specific of T cells (*p* = 0.0003), CD8^+^ T cells (*p* = 0.01), cytotoxic lymphocytes (*p* = 0.0006) and NK cells (*p* = 0.017) (Fig. 2b). To extend these findings to other genes potentially involved in the activity of distinct immune cell subsets, we included known markers of subpopulations of T cells, CD8^+^ T cells, helper T cells, NK cells and B cells (Fig. 2c). Compared to their DC-LAMP^Lo^ counterparts, DC-LAMP^Hi^ tumors exhibited an overrepresentation of gene sets specifically clustering to the following immunological functions: T cells, CD8 cytotoxicity, T_H_1 polarization, T cell activation, NK cells and plasma cells (Fig. 2c). The expression of genes related to T_H_2 polarization, T cell phenotype and B cells did not differ between DC-LAMP^Hi^ and DC-LAMP^Lo^ samples (Fig. 2c). We next validated these findings by assessing the expression levels of the most significant DEGs in a large group of HGSC patients (*n* = 46). Confirming RNA-Seq data, C-C motif chemokine ligand 5 (*CCL5*), C-C motif chemokine receptor 4 (*CCR4*), *CD3, CD4, CD40, CD40 ligand (CD40L), CD8A,* cytotoxic T-lymphocyte associated protein 4 (*CTLA4*), granzyme A (*GZMA*), granzyme B (*GZMB*), *IL18* and perforin 1 (*PRF1*) were all significantly overrepresented in DC-LAMP^Hi^ HGSCs as compared to their DC-LAMP^Lo^ counterparts (Fig. 2d). Altogether, these results demonstrate that an abundant infiltration by mature DCs correlates with an immune contexture characterized by T_H_1 polarization, infiltration by effectors cells (T cells, NK cells and plasma cells) and cytotoxic effector functions.

### Mature DCs are associated with HGSC infiltration by IFN-γ-producing CD8^+^ T cells

**Fig. 3:**
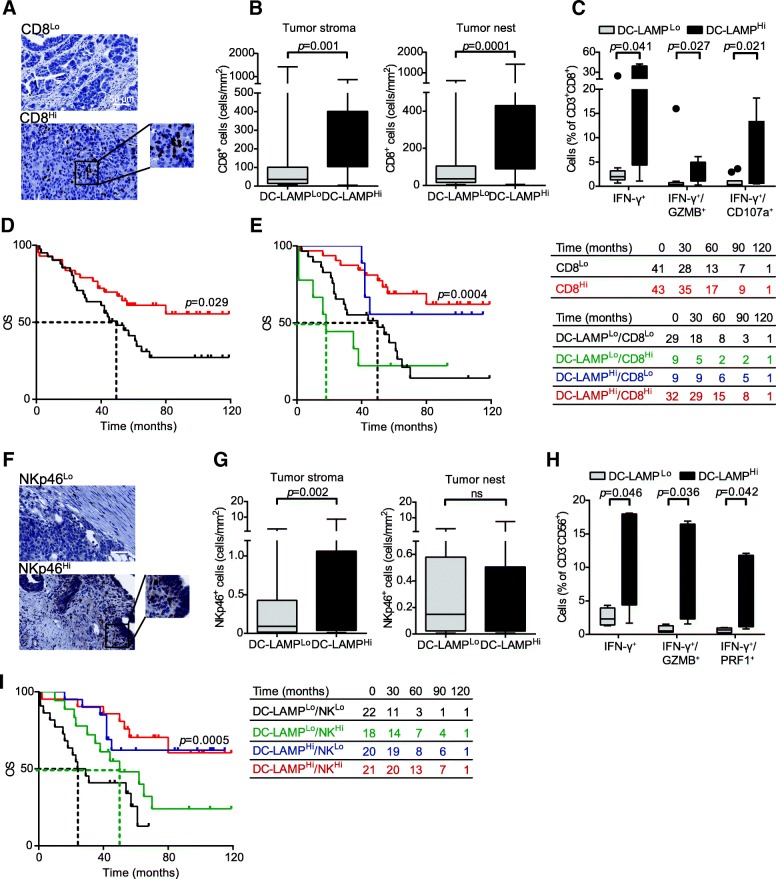
Impact of mature DCs on the frequency and cytotoxicity of CD8^+^ T cells and NK cells in HGSC. **a** Representative images of CD8 immunostaining. Scale bar = 50 μm. **b** Density of CD8^+^ T cells in the tumor stroma and tumor nest in DC-LAMP^Lo^ versus DC-LAMP^Hi^ HGSCs (*n* = 81). Box plots: lower quartile, median, upper quartile; whiskers, minimum, maximum. **c** Percentage of IFN-γ^+^, IFN-γ^+^/GZMB^+^ and IFN-γ^+^/CD107a^+^ cells among CD8^+^CD3^+^ T cells from the HGSC of 10 DC-LAMP^Lo^ and 10 DC-LAMP^Hi^ patients after non-specific stimulation. Box plots: lower quartile, median, upper quartile; whiskers, minimum, maximum. **d, e** OS of HGSC patients who did not receive neoadjuvant chemotherapy, upon stratification based on median density of CD8^+^ cells alone (**d**) or plus DC-LAMP^+^ cells **(e)**. Survival curves were estimated by the Kaplan-Meier method, and differences between groups were evaluated using log-rank test. Number of patients at risk are reported. **f** Representative images of NKp46 immunostaining. Scale bar = 50 μm. **g** Density of NKp46^+^ cells in the tumor stroma and tumor nest in DC-LAMP^Lo^ versus DC-LAMP^Hi^ HGSCs (*n* = 81). Box plots: lower quartile, median, upper quartile; whiskers, minimum, maximum. **h** Percentage of IFN-γ^+^, IFN-γ^+^/GZMB^+^ and IFN-γ^+^/PRF1^+^ cells among CD3^−^CD56^+^ NK cells from the HGSCs of 10 DC-LAMP^Lo^ and 10 DC-LAMP^Hi^ patients after non-specific stimulation. Box plots: lower quartile, median, upper quartile; whiskers, minimum, maximum. **i** OS of HGSC patients who did not receive neoadjuvant chemotherapy, upon stratification based on median density of DC-LAMP^+^ cells plus NKp46^+^ cells. Survival curves were estimated by the Kaplan-Meier method, and differences between groups were evaluated using log-rank test. Number of patients at risk are reported

A correlation between robust tumor infiltration by CD8^+^ T cells and an elevated intratumoral frequency of mature DCs has previously been reported for many types of human cancers [21, 22, 29]. As we observed a positive correlation between DC-LAMP^+^ DC density and the levels of several transcripts associated with CD8^+^ T cell responses, we further evaluated CD8^+^ T cell infiltrate in HGSC samples by IHC (Fig. 3a). We observed a higher density of CD8^+^ T cells in DC-LAMP^Hi^ HGSCs as compared to their DC-LAMP^Lo^ counterparts (*p* = 0.001 and *p* = 0.0001, respectively) (Fig. 3b), corroborating the notion that DC-LAMP^Lo^ lesions have a less abundant infiltrate than DC-LAMP^Hi^ tumors. To address the functional capacity of CD8^+^ T cells found in the HGSC TME, we employed flow cytometry on tumors freshly resected from a prospective cohort of 20 HGSC patients (Additional file 1: Table S5), which were also analyzed for DC-LAMP^+^ cell density by IHC. In line with our previous results, we observed a significantly higher percentage of CD3^+^CD45^+^ and CD3^+^CD8^+^ T cells amongst live mononuclear cells in DC-LAMP^Hi^ versus DC-LAMP^Lo^ HGSCs (Additional file 1: Figure S3B). Moreover, non-specific stimulation caused a more pronounced increase in CD8^+^ T cells staining positively for IFN-γ, IFN-γ/GZMB and IFN-γ/CD107a (CD107a is a marker of degranulation) when CD8^+^ T cells were isolated from DC-LAMP^Hi^ versus DC-LAMP^Lo^ tumors (*p* = 0.041, *p* = 0.027 and *p* = 0.021, respectively) (Fig. 3c, Additional file 1: Figure S3A). These data corroborate our previous findings showing the overrepresentation of *IFNG*, *PRF1* and *GZMB* amongst 23 patients with DC-LAMP^Hi^ HGSC, compared to their 23 DC-LAMP^Lo^ counterparts by qRT-PCR (Fig. 2d). Thus, in both retrospective and prospective cohorts of patients, the presence of mature DCs correlated with increased frequencies of CD8^+^ T cells with enhanced effector functions. Confirming prior observations, high densities of CD8^+^ T cells had a positive impact on the OS of HGSC patients (study group 1) (Fig. 3d, Table 2). Since both mature DCs and CD8^+^ T cells influence disease outcome in patients with HGSC not receiving neoadjuvant chemotherapy, we assessed OS upon stratifying patients from study group 1 into four subsets (DC-LAMP^Hi^/CD8^Hi^, DC-LAMP^Hi^/CD8^Lo^, DC-LAMP^Lo^/CD8^Hi^ and DC-LAMP^Lo^/CD8^Lo^). DC-LAMP^Hi^/CD8^Hi^ patients exhibited the best disease outcome in this setting (median OS > 120 mo.), which was significantly better than OS amongst DC-LAMP^Lo^/CD8^Lo^ patients (median OS: 50 mo., *p* = 0.0004) (Fig. 3e). Intriguingly, DC-LAMP^Lo^/CD8^Hi^ patients had an even poorer median OS (18 mo.) (Fig. 3e). However, the size of this specific patient group was too small to enable a statistical assessment of this observation.

### Mature DCs are associated with HGSC infiltration by cytotoxic NK cells

Both the innate and adaptive arm of the immune system contribute to cancer immunosurveillance [30]. Thus, driven by the positive correlation between the density of mature DCs in the HGSC TME and the abundance of NK cell-related transcripts, we next evaluated the association between DC-LAMP^+^ DCs and NK cells by IHC (based on the specific NK cell marker NKp46) [31]. NK cells were mainly localized to tumor invasive margin and stroma, were rarely in contact with malignant cells, and were generally found outside of TLSs (Fig. 3f). We observed a higher density of NK cells in the tumor stroma (but not in tumor nests) in DC-LAMP^Hi^ versus DC-LAMP^Lo^ samples (Fig. 3g), corroborating RNA-Seq data (Fig. 2c). To obtain insights into the functional capacity of NK cells infiltrating the TME, we used flow cytometry on freshly resected samples from a prospective cohort of 20 patients with HGSC (Additional file 1: Table S5), which were also analyzed for DC-LAMP^+^ cell density by IHC (Additional file 1: Figure S3C). We next compared the expression of 15 NK cell markers amongst CD3^−^CD56^+^ NK cells isolated from freshly resected DC-LAMP^Hi^ or DC-LAMP^Lo^ HGSCs. Although NK cell receptors were not differentially expressed in these two groups of samples (data not shown), non-specific stimulation was much more effective at inducing the acquisition of effector functions amongst NK cells from DC-LAMP^Hi^ patients (versus their DC-LAMP^Lo^ counterparts), as assessed by the percentage of NK cells expressing IFN-γ, IFN-γ/GZMB and IFN-γ/PRF1 (*p* = 0.046, *p* = 0.036 and *p* = 0.042, respectively) (Fig. 3h). These results suggest that intratumoral NK cells from DC-LAMP^Lo^ patients display an impaired capacity to acquire effector functions, even though their surface phenotype is unaltered.

Although NK cells have been associated with improved disease outcome in patients with some solid tumors [22], their prognostic impact on HGSC is unknown. We therefore stratified HGSC patients based on median NKp46^+^ cell density in the TME, finding no significant differences in OS between these two groups (Additional file 1**:** Figure S3D**,** Table 2). Importantly, when we stratified patients into four groups based on both DC-LAMP^+^ cell density and NKp46^+^ NK cell density, we observed that DC-LAMP^Hi^/NKp46^Hi^ patients had superior disease outcome (median OS > 120 mo.) as compared to DC-LAMP^Lo^/NKp46^Lo^ patients (median OS 26.5 mo.; *p* = 0.0005) (Fig. 3i).

### CD20^+^ B cells in the HGSC TME correlate with mature DCs and long-term survival

**Fig. 4:**
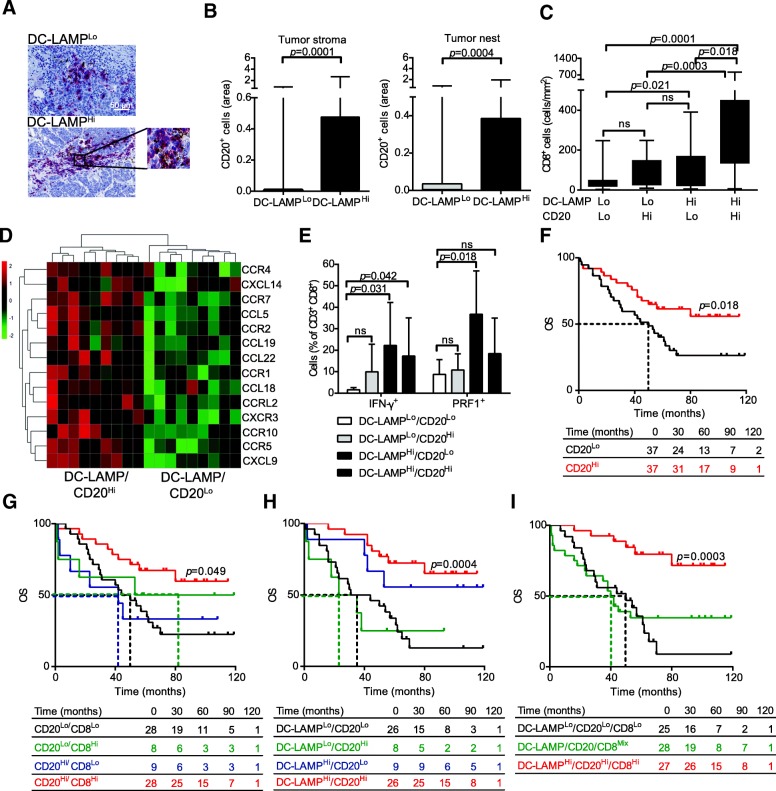
Impact of mature DCs and CD20^+^ B cells on the immune contexture of HGSCs and associated anticancer immune response. **a** Representative images of DC-LAMP immunostaining. Scale bar = 50 μm. **b** Density of CD20^+^ B cells in the tumor stroma and tumor nest in DC-LAMP^Lo^ versus DC-LAMP^Hi^ HGSCs (*n* = 81). Box plots: lower quartile, median, upper quartile; whiskers, minimum, maximum. **c** Density of CD8^+^ T cells in DC-LAMP^Lo^/CD20^Lo^, DC-LAMP^Lo^/CD20^Hi^, DC-LAMP^Hi^/CD20^Lo^, DC-LAMP^Hi^/CD20^Hi^ HGSCs. Box plots: lower quartile, median, upper quartile; whiskers, minimum, maximum. **d** Relative expression levels of *CCR4, CXCL14, CCR7, CCL5, CCR2, CCL19, CCL22, CCR1, CCL18, CCRL2, CXCR3, CCR10, CCR5* and *CXCL9* in 9 DC-LAMP^Hi^/CD20^Hi^ versus 9 DC-LAMP^Lo^/CD20^Lo^ HGSCs, as determined by RNA-Seq and IHC. Benjamin-Hochberg correction was used for RNA-Seq data. **e** Percentage of IFN-γ^+^ and PRF1^+^ cells among CD8^+^CD3^+^ T cells from 10 DC-LAMP^Lo^ and 10 DC-LAMP^Hi^ samples. Box plots: lower quartile, median, upper quartile; whiskers, minimum, maximum. **f, g, h, i** OS of HGSC patients who did not receive neoadjuvant chemotherapy, upon stratification based on median density of CD20^+^ cells plus CD8^+^ cells (**g**), CD20^+^ cells plus DC-LAMP^+^ cells (**h**), or median density of all 3 parameters (**i**). Survival curves were estimated by the Kaplan-Meier method, and differences between groups were evaluated using log-rank test. Number of patients at risk are reported

B cell density strongly correlates with CD8^+^ T cell infiltration and favorable disease outcome in patients with HGSC [13]. Nevertheless, the association between CD20^+^ cells and mature DCs, as well as their impact on the immune contexture of HGSC remain unknown. Tumor-infiltrating CD20^+^ B cells were found in > 50% of patients with HGSC by IHC (data not shown), exhibiting a robust positive correlation with DC-LAMP^+^ DC density, in both the tumor stroma and tumor nests (*p* = 0.0001 and *p* = 0.0004, respectively) (Fig. 4a, b). These results are in line with our previous findings showing that a high density of DC-LAMP^+^ cells in the TME is associated with the overrepresentation of a plasma cell gene signature (Fig. 2c). Next, we divided our entire patient cohort into 4 groups according to the density of mature DCs and CD20^+^ B cells (DC-LAMP^Lo^/CD20^Lo^, DC-LAMP^Lo^/CD20^Hi^, DC-LAMP^Hi^/CD20^Lo^, DC-LAMP^Lo^/CD20^Lo^), with the specific aim to assess CD8^+^ T cell infiltration. Interestingly, we observed a significantly higher density of CD8^+^ T cells in DC-LAMP^Hi^/CD20^Hi^ patients as compared to all other groups. Moreover, DC-LAMP^Hi^/CD20^Lo^ samples exhibited a significantly higher density of CD8^+^ T cells than DC-LAMP^Lo^/CD20^Lo^ group (Fig. 4c). By combining IHC and biomolecular analyses, we demonstrated that *CCR4, CXCL14, CCR7, CCL5, CCR2, CCL19, CCL22, CCR1, CCL18, CCRL2, CXCR3, CCR10, CCR5* and *CXCL9* are overrepresented in DC-LAMP^Hi^/CD20^Hi^ samples as compared their DC-LAMP^Lo^/CD20^Lo^ counterparts (Fig. 4d). Functional analyses revealed that all these DEGs are mainly involved in lymphocyte, DC and monocyte chemotaxis (Additional file 1**:** Figure S4). Finally, by combining IHC and flow cytometry, we detected a significantly higher percentage of IFN-γ^+^ and PRF1^+^ cells amongst CD8^+^ T cells from DC-LAMP^Hi^/CD20^Lo^ tumors compared to their DC-LAMP^Lo^/CD20^Lo^ counterparts (Fig. 4e).

Confirming prior observations [15], robust tumor infiltration by CD20^+^ B cells had a positive impact on the survival of patients with HGSC (*p* = 0.018) (Fig. 4f, Table 2). Importantly, high levels of both CD8^+^ CTLs and CD20^+^ B cells were associated with most favorable clinical outcome amongst the patients involved in this study (*p* = 0.049) (Fig. 4g). Indeed, median survival for CD8^Hi^/CD20^Hi^ patients was > 120 mo., where it was only 47 mo. for CD8^Lo^/CD20^Lo^ patients (*p* = 0.0004). These findings are in accordance with the results from survival analysis showing that DC-LAMP^Hi^ patients have the best OS irrespective of CD20^+^ B cell density (Fig. 4h). Altogether, our data suggest that while both mature DC-LAMP^+^ DCs and CD20^+^ B cells shape the immune contexture of HGSCs, only the former are critical for enabling a clinically relevant anticancer response driven by CD8^+^ CTLs.

Finally, we evaluated the combined prognostic value of DC-LAMP^+^, CD8^+^ and CD20^+^ cell density by stratifying our cohort in three groups of patients: DC-LAMP^Hi^/CD8^Hi^/CD20^Hi^ patients, DC-LAMP^Lo^/CD8^Lo^/CD20^Lo^ patients, and patients in which either of the three parameters was discordant with the remaining two (which we named DC-LAMP/CD8/CD20^Mix^). DC-LAMP^Hi^/CD8^Hi^/CD20^Hi^ patients had superior RFS (not shown) and OS (median > 120 mo.) compared with their DC-LAMP^Lo^/CD8^Lo^/CD20^Lo^ counterparts (median OS: 47 mo.) as well as DC-LAMP/CD8/CD20^Mix^ patients (median OS 40 mo.) (Fig. 4i). Taken together, these findings indicate that the concomitant assessment of DC-LAMP^+^ DCs, CD20^+^ B cells and CD8^+^ CTLs in the TME of patients with HGSC conveys robust prognostic information.

## Discussion

The composition of the immune infiltrate in human solid tumors, its localization and functional orientation are major predictors of patient survival, as previously documented [5, 32]. In particular, high densities of intratumoral CD8^+^ CTLs and CD20^+^ B cells have been associated with improved clinical outcome in patients affected by a variety of tumors, including HGSC [13, 21]. Elevated intratumoral levels of DC-LAMP^+^ DCs also constitute a robust positive prognostic value in multiple oncological settings, including non-small-cell lung carcinoma (NSCLC) [14, 21, 33], melanoma [34], renal cell carcinoma (RCC) [29], breast cancer [35] and colorectal carcinoma (CRC) [22]. Nevertheless, the impact of DC-LAMP^+^ cells on the composition and functional orientation of the immune contexture of HGSC and its prognostic role remained to be elucidated. Here, we assessed the prognostic value of DC-LAMP^+^ cell densities in two independent retrospective cohorts of patients with HGSC who did not receive neoadjuvant chemotherapy (*n* = 81 and *n* = 66).

We observed a major inter-individual variability in DC-LAMP^+^ cell densities. The great majority of mature DCs were localized in the tumor stroma and associated with TLSs (rather than being in direct contact with malignant cell nests), as previously observed in samples from NSCLC, RCC and CRC [14, 21, 22, 29]. In NSCLCs, mature DCs provide a specific marker for TLSs and constitute a favorable prognostic biomarker for survival [21, 33]. TLSs were identified only in 19% of our HGSC samples, which is in line with previous findings, and their abundance did not correlate with OS [15]. We therefore decided to evaluate the prognostic impact of DC-LAMP^+^ DCs in the entire tumor stroma and tumor nest. High densities of mature DCs in the TME were strongly associated with improved RFS and, most importantly, superior OS in both independent retrospective cohorts.

By combining IHC and biomolecular analyses, we demonstrated that a high density of tumor-infiltrating mature DCs is associated with a T_H_1-polarized immune contexture that acquired effector functions. These results recapitulate previous findings in the setting of NSCLC [21, 33] and CRC [22, 36]. The presence of tumor-infiltrating CD8^+^ CTLs is strongly associated with improved clinical outcome amongst patients with HGSC [9, 12, 13]. Accordingly, we found a strong correlation between CD8^+^ CTL density and improved OS in our cohorts of HGSC patients. In both prospective and retrospective studies, we showed that CD8^+^ T cells co-localize preferentially with mature DCs in the HGSC TME, and that the density of CD8^+^ T cells is profoundly diminished in DC-LAMP^Lo^ tumors. DC-LAMP^Hi^ patients with a concomitantly elevated density of CD8^+^ CTLs in their tumors had a significant clinical benefit as compared to patients with low intratumoral levels of both mature DCs and CD8^+^ CTLs. Moreover, DC-LAMP^Lo^/CD8^Hi^ patients had significantly worse disease outcome than their DC-LAMP^Hi^/CD8^Hi^ counterparts. Thus, DC-LAMP stands out as a robust biomarker allowing for the identification of CD8^Hi^ HGSC patients with higher risk of death.

In contrast to the well-established antitumor activity of CD8^+^ CTLs, there is little evidence in support of a similar function from NK cells (in the setting of HGSC) [37]. NKp46^+^ NK cells were mainly localized at invasive tumor margins and within the stroma of HGSC samples, which is in line with previous finding in the NSCLC, CRC and RCC setting [22, 25]. Importantly, increased intratumoral levels of NK cells have been associated with good prognosis in patients with RCC, although a similar prognostic value could not be documented in NSCLC and CRC [22, 25]. Along similar lines, the density of NK cells did not influence clinical outcome in our cohorts of HGSC patients. Although the cell surface properties of NK cells did not differ between DC-LAMP^Hi^ and DC-LAMP^Lo^ samples, we observed significantly a higher frequency of IFN-γ^+^/PRF1^+^ and IFN-γ^+^/GZMB^+^ NK cells after non-specific stimulation in the former. Finally, in line with previous observations on CRC, DC-LAMP^Hi^ patients with concomitantly elevated amounts of intratumoral NK cells had a significant clinical benefit compared with DC-LAMP^Lo^/NKp46^Lo^ individuals [22]. Taken together, our data demonstrate that robust tumor infiltration by mature DCs generates an immune contexture characterized by T_H_1 polarization and cytotoxic functions.

Importantly, the presence of CD20^+^ B cells correlates with improved OS in our cohorts of chemotherapy-naive patients with HGSC, which is line with previous observations from other oncological settings [13, 14, 38–41]. Notably, HGSCs containing elevated amounts of both CD8^+^ CTLs and CD20^+^ B cells are associated with superior survival than HGSCs containing high levels of either CD8^+^ CTLs or CD20^+^ B cells [13]. These findings suggest not only the existence of cooperative interactions between CD8^+^ CTLs and CD20^+^ B cells in the TME of HGSCs, but also the critical role of B cells in regulation of the immune infiltrate, as previously reported in a variety of other cancers [42, 43]. CD8^+^ CTLs and CD20^+^ B cells often co-localize in lymphoid aggregates of various sizes and morphology in HGSC samples, as previously described in detail [15]. In all such aggregates, especially in TLSs, B cells form follicles adjacent to discrete zones containing not only CD4^+^ and CD8^+^ T cells, but also high densities of DCs [18]. Accordingly, we identified a robust correlation between the presence of DC-LAMP^+^ DCs and CD20^+^ B cells in the TME of HGSC lesions.

In conclusion, by combining IHC and biomolecular analyses, we comprehensively documented for the first time the influence of both mature DCs and CD20^+^ B cells on the establishment of the immune contexture of HGSC. That said, DCs stand out as the critical attribute for the initiation of an immune response to HGSC which exhibits T_H_1 polarization, is armed with immune effectors, and mediates clinical benefits. These findings are in line with previous studies documenting the critical role of mature DCs in the activation of antitumor immunity [44, 45]. Accordingly, we identified tumor infiltration by DC-LAMP^+^ DCs as a robust, positive prognostic biomarker for HGSC patients, as confirmed by both univariate and multivariate analyses.

## Additional file

Additional file 1: **Figure S1.** Density of mature DCs in the tumor stroma and malignant cell nest of patients with HGSCs. **(A)** Representative images of DC-LAMP immunostaining (in brown) and CD20 immunostaining (in red) are shown. Scale bar = 50 μm. **(B)** Density of DC-LAMP^+^ cells in the tumor stroma and nest of patients with HGSCs (*n* = 81). **(C)** RFS and OS of 81 patients with HGSC who did not receive neoadjuvant chemotherapy, upon stratification based on median density of DC-LAMP^+^ cells in the tumor nest. **Figure S2.** Prognostic impact of tertiary lymphoid structures in HGSC patients. OS of 147 patients with HGSC who did not receive neoadjuvant chemotherapy, upon stratification based on presence or absence of TLSs. **Figure S3.** Evaluation of the functional profile of CD8^+^ T cells and NK cells from DC-LAMP^Hi^ versus DC-LAMP^Lo^ HGSC samples. **(A)** Gating strategy for CD8^+^ T cells. The percentage of cells in each gate is reported. **(B)** Percentage of CD45^+^CD3^+^ cells and CD3^+^CD8^+^ cells from freshly resected DC-LAMP^Hi^ (*n* = 10) and DC-LAMP^Lo^ (*n* = 10) HGSCs. Boxplots: lower quartile, median, upper quartile; whiskers, minimum, maximum. **(C)** Gating strategy for NK cells. The percentage of cells in each gate is reported. **(D)** OS of 81 patients with HGSC who did not receive neoadjuvant chemotherapy, upon stratification based on median NK cell density. **Figure S4.** ClueGo analysis of genes overrepresented in DC-LAMP^Hi^/CD20^Hi^ versus DC-LAMP^Lo^/CD20^Lo^ HGSCs. **Table S1.** Main clinical and biological characteristics of 66 HGSC patients enrolled in the validation cohort (University Hospital Motol). **Table S2.** The list of antibodies used for IHC staining. **Table S3.** The list of antibodies used for flow cytometry. **Table S4.** List of genes significantly overrepresented in DC-LAMP^Hi^ versus DC-LAMP^Lo^ HGSC samples as per RNA-Seq. **Table S5.** Main clinical and biological characteristics of 20 HGSC patients in which the freshly resected tumors were analyzed using flow cytometry (University Hospital Motol). **Table S6.** Main clinical and biological characteristics of 18 HGSC patients whose tumor samples were used for NGS data analysis (University Hospital Motol). (PDF 785 kb)
