# Supplementary material for: Mature dendritic cells correlate with favorable immune infiltrate and improved prognosis in ovarian carcinoma patients
Source: J Immunother Cancer. 2018 Dec 4;6:139. doi: 10.1186/s40425-018-0446-3 (PMC6288908; doi:10.1186/s40425-018-0446-3)
Supplement: Supplementary file 1 — Figure S1. Density of mature DCs in the tumor stroma and malignant cell nest of patients with HGSCs. (A) Representative images of DC-LAMP immunostaining (in brown) and CD20 immunostaining (in red) are shown. Scale bar = 50 μm. (B) Density of DC-LAMP+ cells in the tumor stroma and nest of patients with HGSCs (n = 81). (C) RFS and OS of 81 patients with HGSC who did not receive neoadjuvant chemotherapy, upon stratification based on median density of DC-LAMP+ cells in the tumor nest. Figure S2. Prognostic impact of tertiary lymphoid structures in HGSC patients. OS of 147 patients with HGSC who did not receive neoadjuvant chemotherapy, upon stratification based on presence or absence of TLSs. Figure S3. Evaluation of the functional profile of CD8+ T cells and NK cells from DC-LAMPHi versus DC-LAMPLo HGSC samples. (A) Gating strategy for CD8+ T cells. The percentage of cells in each gate is reported. (B) Percentage of CD45+CD3+ cells and CD3+CD8+ cells from freshly resected DC-LAMPHi (n = 10) and DC-LAMPLo (n = 10) HGSCs. Boxplots: lower quartile, median, upper quartile; whiskers, minimum, maximum. (C) Gating strategy for NK cells. The percentage of cells in each gate is reported. (D) OS of 81 patients with HGSC who did not receive neoadjuvant chemotherapy, upon stratification based on median NK cell density. Figure S4. ClueGo analysis of genes overrepresented in DC-LAMPHi/CD20Hi versus DC-LAMPLo/CD20Lo HGSCs. Table S1. Main clinical and biological characteristics of 66 HGSC patients enrolled in the validation cohort (University Hospital Motol). Table S2. The list of antibodies used for IHC staining. Table S3. The list of antibodies used for flow cytometry. Table S4. List of genes significantly overrepresented in DC-LAMPHi versus DC-LAMPLo HGSC samples as per RNA-Seq. Table S5. Main clinical and biological characteristics of 20 HGSC patients in which the freshly resected tumors were analyzed using flow cytometry (University Hospital Motol). Table S6. Main clinical and bi [file 40425_2018_446_MOESM1_ESM.pdf]

Supplemental information

Supplemental Material and Methods

Isolation of mononuclear cells from the frest ovarian tumor specimens

Fresh ovarian tumor specimens were minced with scissors, digested in PBS containing 1 mg/ml of Collagenase D (Roche) and 0,2mg/ml DNase I at 37°C for 30 min, mechanically dissociated using the gentleMACS dissociator (Miltenyi Biotec) and passed through a 100-µm nylon cell strainer (BD Biosciences).

RNA extraction and reverse transcription

RNA was isolated from FFPE tissue sections using the AllPrep DNA/RNA FFPE Kit (Qiagen) following the manufacturer's instructions. Briefly, three 10 µm-thick scrolls of FFPE were cut from each paraffin block and deparaffinized using xylene. Total nucleic acids were released from the tissue by protease K digestion (included in the isolation kit) at 56°C/15 min. The RNA-containing supernatant was incubated at 80°C/15 min to partially reverse formalin crosslinking, followed by RNA purification using RNeasy MinElute spin columns and chemistry, which was included in the kit. Residual genomic DNA was removed from the total RNA by DNase treatment.

The concentration and purity of the samples was assessed by spectrophotometry (Nanodrop 2000c; Thermo Scientific), and the RNA integrity was quantified on a Bioanalyzer 2100 instrument (Agilent). The RNA integrity was variable across samples as expected for FFPE samples. cDNA synthesis was performed using a TATAA GrandScript cDNA Synthesis Kit (TATAA Biocenter). Briefly, 3 µl 5X TATAA GrandScript RT Reaction Mix, 0.75 µl TATAA GrandScript RT Enzyme, 1.25 µl water and 10 µl total RNA (100 ng/µl) were added

to a final volume of 15 µl. The following thermal program was used: 25°C for 5 min, 42°C for 30 min and 85°C for 5 min. The samples were stored at –20°C.

cDNA preamplification

Ten microliters of 2-times diluted cDNA was used in a 50-µl preamplification reaction using a TATAA PreAmp GrandMaster® Mix and 40 nM each primer. The following thermal profile for targeted preamplification was applied on a T100 Thermal Cycler (Bio-Rad): 95°C for 3 min, followed by 20 cycles of amplification (95°C for 20 s, 55°C for 3 min and 72°C for 20 s). After the final extended (10 min) elongation step, the samples were immediately frozen and stored at –20°C until analysis on the BioMark high-throughput qPCR platform (Fluidigm).

High-throughput quantitative real-time PCR

High-throughput qPCR was performed on the BioMark system using the 48.48 Dynamic Array Chip for Gene Expression and probe-based detection. Each 5-µl sample reaction contained 1 µl of 10 times diluted preamplification product, 2.74 µl Probe GrandMaster Mix (TATAA Biocenter), 0.25 µl 20X GE Sample Loading Reagent (Fluidigm), 0.01 µl ROX (Life Technologies, the final concentration in the sample mix was 50 nM) and DNA-free water. The 5-µl assay reaction mix contained 2.5 µl Assay Loading Reagent (Fluidigm) and 2.5 µl of a 5 µM mix of reverse and forward primers and 2.5 µM probes. Priming and loading of the dynamic array were performed according to the manufacturer´s instructions using the IFC controller HX. The temperature profile contained: thermal mixing at 50°C for 2 min followed by 70°C for 40 min and 25°C for 10, followed by hot start activation at 95°C for 30 s and 40 cycles of amplification (95°C for 10 s and 60°C for 60 s). Melting curve analysis was performed in the range of 60°C to 95°C at 0.5°C per second increments. Amplification data

were analyzed with Fluidigm Real-Time PCR Analysis software, applying the linear derivative baseline subtraction method and a user-defined global threshold to obtain Cq-values. Melting curve analysis was performed on all the samples. Preamplification and Biomark´s measurement were performed at BIOCEV Gene Core Facility (Vestec, Prague).

Library preparation and sequencing

Twenty FFPE samples suitable for library preparation according to quality/quantity evaluations were processed following the manufacturer's specifications with minor modifications by using the Illumina TruSeq® RNA Access Library Prep (Illumina) along with purification steps employing SPRI beads. DV200 values for all samples exceeded 40%, and 100 ng of total RNA was used for the cDNA synthesis. Each library was quantified with the fluorimeter Qubit 2.0 (dsDNA HS kit; Thermo Fisher), and the size distribution was determined using a DNA 1000 kit on a 2100 Bioanalyzer instrument prior to pooling. All libraries had a similar size distribution of approximately 260 bp. A 4-plex pool of libraries was made by combining 200 ng of each DNA library. The libraries were sequenced on an Illumina NextSeq 500. At least 180 M pair-end 2x75 bp reads were generated per library. The libraries were prepared and sequenced at EMBL Genomics Core Facility (Heidelberg, Germany).

NGS data processing

The raw FASTQ sequencing files were aligned to human reference genome (build h19) with bowtie2 (version 2.3.2) and tophat2 (version 2.1). The levels of expression as raw "counts" were calculated from aligned reads with mapping quality at least 10 using htseq-count (version 0.6.0). Count files were transferred to the GALAXY environment (Galaxy version

18.01), and differentially expressed genes (DEGs) between groups were determined using DEseq2 (Galaxy Version 2.11.40.1) with default settings (Love et al, Genome Biology, 2014).

Supplemental Figures

Supplemental Figure 1

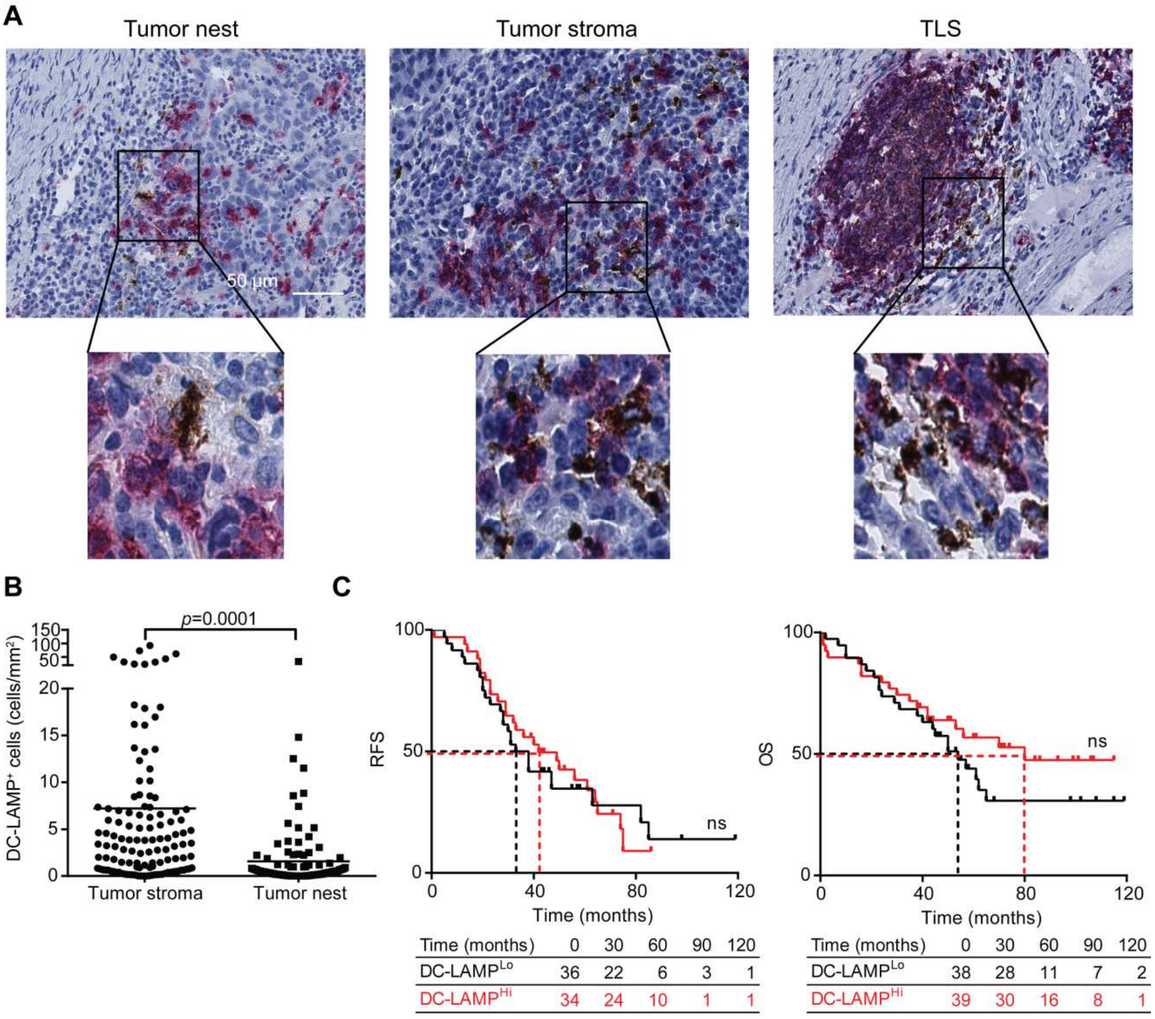

Supplemental Figure 1. **Density of mature DCs in the tumor stroma and malignant cell nest of patients with HGSCs.**

(A) Representative images of DC-LAMP immunostaining (in brown) and CD20 immunostaining (in red) are shown. Scale bar = 50 µm.

(B) Density of DC-LAMP$^+$ cells in the tumor stroma and tumor nest of patients with HGSCs (n=81).

(C) RFS and OS of HGSC patients (n=70 and n=77, respectively) who did not receive neoadjuvant chemotherapy, upon stratification based on median density of DC-LAMP$^+$ cells in the tumor nest. Survival curves were estimated by the Kaplan-Meier method, and differences between groups were evaluated using log-rank test. Number of patients at risk are reported.

Supplemental Figure 2

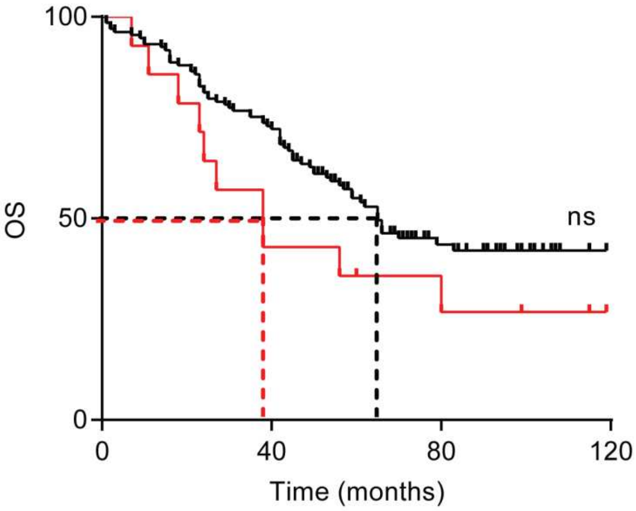

Time (months)	0	30	60	90	120
TLS ^{Neg}	133	104	53	22	1
TLS ^{Pos}	14	9	5	4	1

***Supplemental Figure 2*. Prognostic impact of tertiary lymphoid structures in HGSC patients.**

OS of 147 patients with HGSC who did not receive neoadjuvant chemotherapy, upon stratification based on presence or absence of TLSs. Survival curves were estimated by the

Kaplan-Meier method, and differences between groups were evaluated using log-rank test. Number of patients at risk are reported.

Supplemental Figure 3

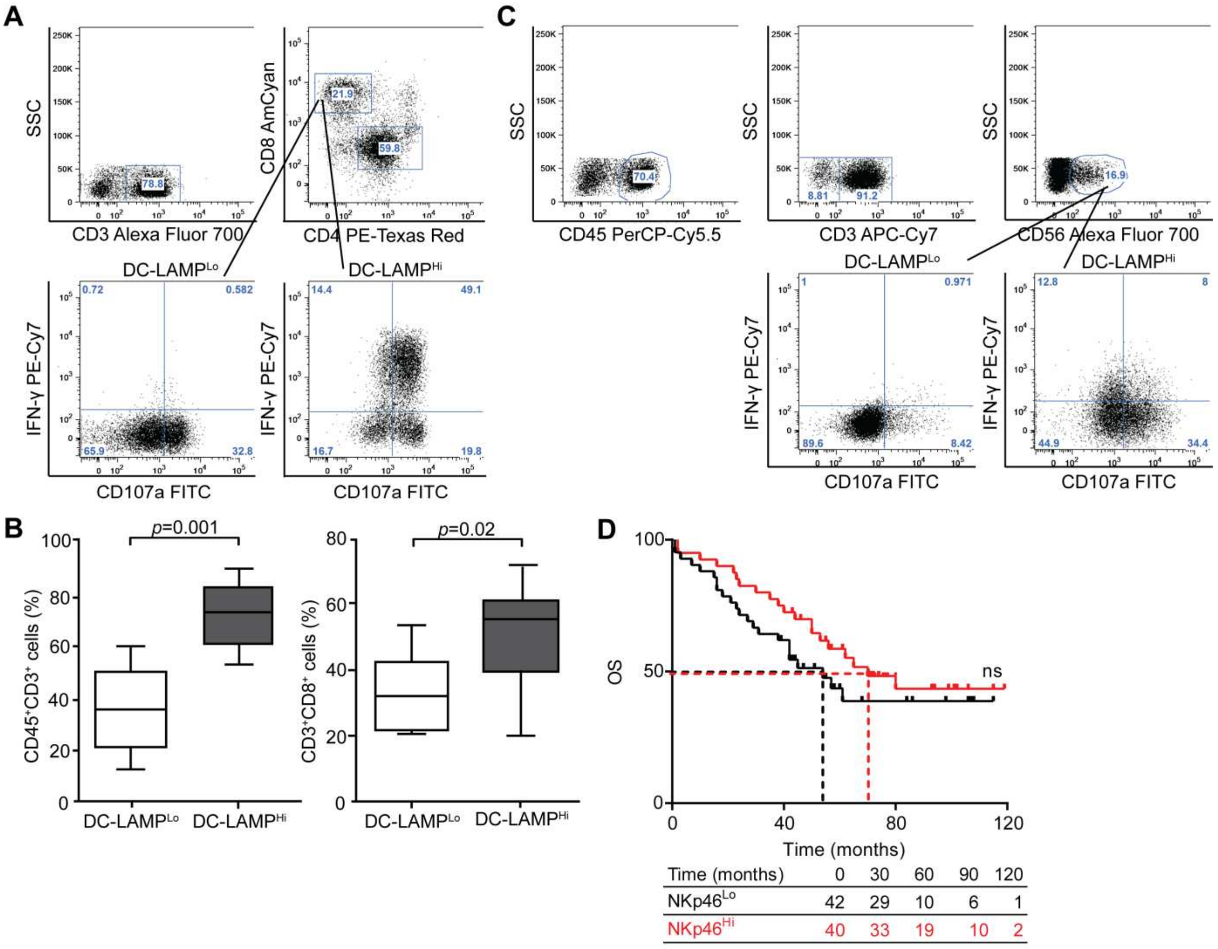

Supplemental Figure 3. **Evaluation of the functional profile of CD8$^+$ T cells and NK cells from DC-LAMP^{Hi} versus DC-LAMP^{Lo} HGSC samples.**

(A) Gating strategy for CD8$^+$ T cells. The percentage of cells in each gate is reported.

(B) Percentage of CD45$^+$CD3$^+$ cells and CD3$^+$CD8$^+$ cells from freshly resected DC-LAMP^{Hi} (n=10) and DC-LAMP^{Lo} (n=10) HGSCs. Boxplots: lower quartile, median, upper quartile; whiskers, minimum, maximum.

(C) Gating strategy for NK cells. The percentage of cells in each gate is reported.

(D) OS of 82 patients with HGSC who did not receive neoadjuvant chemotherapy, upon stratification based on median NK cell density. Survival curves were estimated by the Kaplan-Meier method, and differences between groups were evaluated using log-rank test. Number of patients at risk are reported.

Supplemental Figure 4

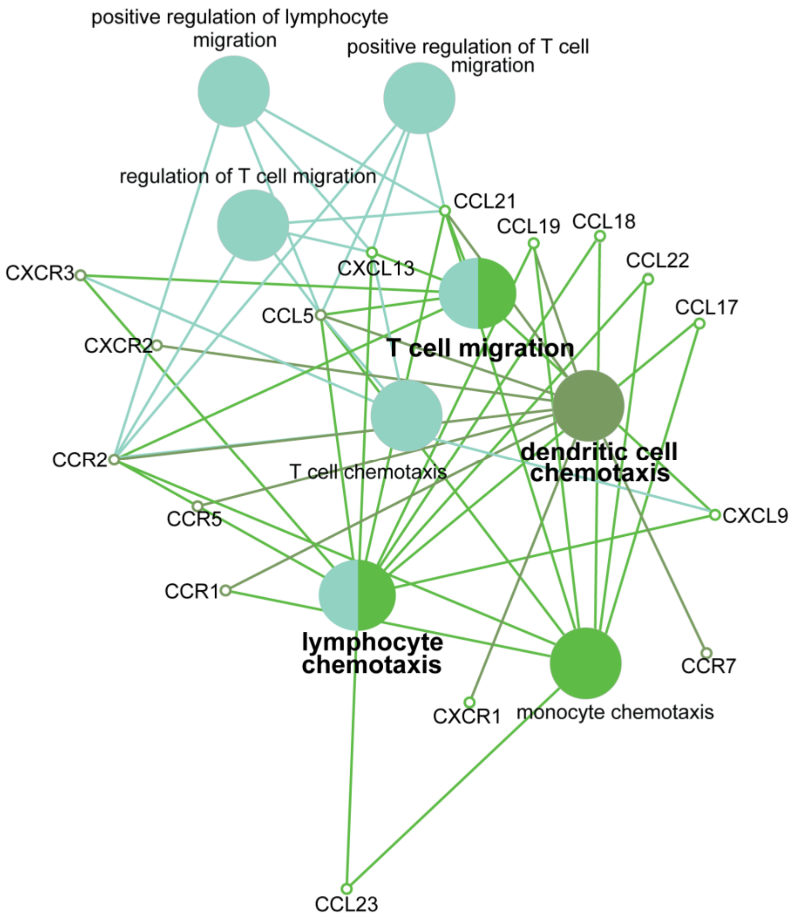

Supplemental figure 4. **ClueGo analysis of genes overrepresented in DC-LAMP^{Hi}/CD20^{Hi} versus DC-LAMP^{Lo}/CD20^{Lo} HGSCs.**

Supplemental Tables

Supplemental Table 1

Variable	Overall cohort (n=66)
**Mean age at diagnosis ±SEM**	58.53±1.38
**Stage of diasease:**	
Stage I	0 (0%)
Stage II	3 (4,5%)
Stage III and IV	63 (95,5%)
**Debulking**	
R0	30 (45,5%)
R1	7 (10,5%)
R2	29 (44,0%)
**Vital status of patients**	28 (41,7%)

***Supplemental Table 1*. Main clinical and biological characteristics of 66 HGSC patients enrolled in the validation cohort (University Hospital Motol)**

Supplemental Table 2

Parameter	Source	Producer	Clone	Detection system	Revelation	Dilution	Incubation time [min]
**CD20**	mouse	Dako	L26	ImmPRESS-AP anti-mouse IgG (alkaline phosphatase) Polymer Detection Kit	ImmPACT Vector red Alkaline Phosphatase substrate kit	1:250	60
**CD8**	rabbit	Spring Bioscience	SP16	EnVision™+/HRP, Rabbit	DAB+ substrate Chromogen system	1:80	30
**DC-LAMP**	rat	Dendritics	1010E1.01	donkey anti-rat IgG-biot (Jackson ImmunoResearch)	DAB+ substrate Chromogen system	1:80	60
**NKp46**	mouse	RD systems	195314	Impress HRP anti-mouse IgG (Peroxidase) Polymer Detection kit	DAB+ substrate Chromogen system	1:100	90

***Supplemental Table 2*. The list of antibodies used for IHC staining.**

Supplemental Table 3

Parameter	Source	Producer	Clone	Fluorochrome	Dilution
**CCR7**	mouse	BioLegend	G043H7	PerCP-Cy5.5	6:100
**CD3**	mouse	EXBIO	MEM-57	Alexa 700	5:100
**CD3**	mouse	eBioscience	OKT3	APC-eFluor780	2:100
**CD3**	mouse	BD Biosciences	SK7	AmCyam	6:100
**CD4**	mouse	eBioscience	RPA-T4	PE-Cy7	5:100
**CD4**	mouse	Beckman Coulter	SFCI12T4D11	ECD	5:100
**CD8**	mouse	BD Biosciences	RPA-T8	HV500	5:100
**CD16**	mouse	BioLegend	3G8	PB	6:100
**CD45**	mouse	Life technologies	HI30	PE-Texas Red	6:100
**CD45**	mouse	EXBIO	MEM-28	PerCP	6:100
**CD45RO**	mouse	EXBIO	UCHL1	APC	6:100
**CD56**	mouse	BioLegend	HCD56	A700	2:100
**CD56**	mouse	BioLegend	HCD56	BV421	6:100
**CD56**	mouse	BioLegend	MEM-188	PerCP-Cy5.5	6:100
**CD62L**	mouse	EXBIO	DREG56	FITC	5:100
**CD69**	mouse	BD Biosciences	FN50	PE-Texas Red	5:100
**CD69**	mouse	Beckman Coulter	TP1.55.3	ECD	6:100
**CD107a**	mouse	BioLegend	H4A3	FITC	8:100
**CD158a.b2.j**	mouse	Beckman Coulter	GL183	PE	6:100
**CD158a.h**	mouse	eBioscience	HP-MA4	APC	6:100
**CD158e1**	mouse	BioLegend	DX9	BV421 (PB)	6:100
**CD227**	mouse	BD Biosciences	HMPV	FITC	2:100
**DNAM-1**	mouse	BioLegend	11A8	PE	6:100
**Epitelial antigen**	mouse	DAKO	Ber-EP4	FITC	2:100
**Epcam**	mouse	BioLegend	9C4	FITC	2:100
**Granzyme**	mouse	BD Biosciences	GB11	BV421	4:100
**IFNg**	murine	eBioscience	4S.B3	PE-Cy7	1:100
**ILT2T/CD85j**	mouse	BioLegend	GHI/75	PE-Cy7	6:100
**Pan cytokeratin**	murine	eBioscience	AE1/AE3	A488	2:100
**Perforin**	mouse	BioLegend	dG9	APC	4:100
**NKG2A/CD159a**	mouse	RD systems	# 705829	A700	6:100
**NKG2D**	mouse	BioLegend	1D11	PE-Cy7	6:100
**NKp30**	mouse	BioLegend	P30-15	APC	6:100
**NKp46**	mouse	BioLegend	9E2	A700	6:100
**NKp80**	mouse	Miltenyi Biotec		FITC	6:100

Supplemental table 3. **The list of antibodies used for flow cytometry.**

Supplemental Table 4

Gene symbol	Base mean	log2(FC)	StdErr	Wald-Stats	P-value	P-adj
CDH18	261.258816032027	-3,514402376	0.509146720633025	-6.90253365869627	5.10831131069219e-12	6.39994782560071e-08
ZNF729	624.125849859806	-2,952605811	0.536589017225152	-5.50254611273777	3.74345271277349e-08	0.000117249618279957
ZNF723	106.221097959686	-2,427222761	0.548595652655835	-4.42442944857769	9.66974972048116e-06	0.00481748164212982
CRYGB	4.20575942381116	-2,351119611	0.539223802115771	-4.36019256092087	1.2994803935722e-05	0.0055188271562269
RP11-760D2.7	6.39000483914034	-2,318369595	0.547880755130456	-4.23152223018061	2.32115048149511e-05	0.00775480901530973
FGF17	62.7349136621099	-2,317588597	0.535575955621662	-4.32728275568063	1.50960117133651e-05	0.00600413913494903
ZNF679	97.6707719209358	-2,301095104	0.5495799122801	-4.18700729955306	2.82656738514374e-05	0.00823549988017984
IL9RP2	2.27446048945075	-2,29755916	0.5502806606652	-4.17524969477609	2.97659382398251e-05	0.00847551266449201
PAGE2	31.0888515257122	-2,273462593	0.549791059124471	-4.13513925886066	3.54739453655151e-05	0.00966163748938818
NPM1P21	4.44586132423718	-2,235869839	0.515142183442781	-4.34029654533143	1.42290560390502e-05	0.00581517759232233
CRYGC	5.52846497591203	-2,196318166	0.532949903097903	-4.1210593217864	3.77134292510724e-05	0.010004092677445
AKR1B15	57.5135592570154	-2,14738494	0.541010029987165	-3.96921465606287	7.21099008949025e-05	0.0164259798793052
DPYSL5	59.1590754960766	-2,1170299	0.544362120687712	-3.8890103113493	0.000100653841920157	0.0203393815886563
RPL34P33	9.65087592603329	-2,114055018	0.487196370019156	-4.3392257169082	1.42985603859556e-05	0.00581517759232233
OACYLP	8.50692647737125	-2,086330359	0.49600999495084	-4.20622644668781	2.59670054245127e-05	0.00793482018197579
DPP10	393.479838366725	-2,06573841	0.525483045516731	-3.93112285415577	8.45500504307131e-05	0.0181074411422425
SNORD115-1	5.84602021435993	-2,026226795	0.550572726650852	-3.6802164300203	0.000233036079133564	0.0326211454460878
LGR5	4491.85316578561	-2,022907997	0.479977244106639	-4.21459146582437	2.50230727846067e-05	0.00793482018197579
ANKRD20A7P	92.4222808612654	-1,964983484	0.536289260495736	-3.66403661061399	0.000248271274843587	0.0332670231751645
DACH1	546.811892429468	-1,947798404	0.444016066113982	-4.38677460726212	1.15043894468075e-05	0.00524119066124827
EXD1	18.3414511890084	-1,94159111	0.513634027279343	-3.78010608032395	0.000156761554963533	0.0258419360705345
ZNF560	41.0693821166824	-1,907227125	0.540022082567548	-3.53175765712045	0.000412807483748884	0.0442981936123293
SIX1	206.506417555358	-1,899778513	0.52909281869063	-3.59063371428269	0.000329874945349015	0.0379159472734416
DMRT3	4.41007380753997	-1,893870775	0.539946990176694	-3.50751242140012	0.000452317236726327	0.0457004556477886
LECT2	8.98353305209725	-1,885808808	0.5178008988909	-3.64195738481074	0.000270572871473347	0.0341925851835221
SCN11A	269.15438107247	-1,880691461	0.474241243933513	-3.96568515581176	7.31854146815879e-05	0.0165207832042932
ZNF99	1715.12300025768	-1,867945804	0.452776307679036	-4.12553787000413	3.69869311580626e-05	0.00996539283900617
TEKT5	14.8373692092618	-1,838924405	0.390617322837707	-4.70773900130206	2.50479521280996e-06	0.00190189859537512
GJB5	8.34103763159188	-1,824984819	0.478920307476426	-3.81062316634427	0.00013861692917825	0.0246335063434001
CTC-451A6.4	19.8881833286579	-1,794425415	0.496208794171242	-3.61627088459099	0.000298877613300441	0.0358324227582255
H3F3BP1	12.3126128337433	-1,784168879	0.492349265751648	-3.62378702029869	0.000290320698134143	0.0350190397801803
SPINT3	10.12974881104	-1,782318975	0.499606242949558	-3.56744736509138	0.000360475757759924	0.039966553372524
SLITRK6	147.019140672917	-1,779521191	0.482529531230213	-3.68790110317763	0.000226111493530432	0.0321913391670002
RP11-121L10.3	90.8586470871601	-1,743978291	0.49627087638464	-3.5141661015154	0.00044113687568035	0.0453015028439448
SLC6A20	38.8061302854927	-1,694484204	0.489498256981372	-3.46167566362368	0.000536823662581909	0.0494529062989518
CLCA4	25.7126981739896	-1,671930298	0.47549258646029	-3.51620686845714	0.000437759754671616	0.0451604201147253
SERBP1P1	6.70278530527558	-1,629938937	0.467985915853338	-3.48288032069771	0.000496050068716071	0.0474351276244829
USP17L6P	977.28707127854	-1,627698488	0.466682172937944	-3.48780943859758	0.000486995035192575	0.0472970333210091

HMGA2	1307.36413827942	-1,619373457	0.420733397178935	-3.84893014837306	0.000118634828086468	0.0222450891515236
PAK7	14.2673624312688	-1,576883861	0.455228616250424	-3.4639383483912	0.000532328498596449	0.0492197608462406
ZNF849P	724.990823398203	-1,549242856	0.409763478470416	-3.78082220045369	0.000156311280379315	0.0258419360705345
CCDC146	2426.26126719839	-1,517924211	0.388411891596088	-3.90802713165987	9.30528586552312e-05	0.01926963206053
HNRNPA1P40	243.265143386932	-1,476308811	0.414248300545895	-3.56382587230886	0.000365488456250187	0.0401218195987808
ALK	899.350878861761	-1,475374093	0.378931275852772	-3.89351364531659	9.88026420852084e-05	0.0201276244124314
RNF144B	910.123448228253	-1,473122893	0.404590063805863	-3.64102587950541	0.000271553835316315	0.0341925851835221
GLRA2	102.286486335371	-1,465544793	0.401200181025324	-3.65290162546157	0.000259293588456142	0.0334903064223998
ERV3-1	1364.4885703965	-1,428385232	0.332557009941979	-4.29515899464821	1.74568180011981e-05	0.00624879269508603
AC139452.2	86.1450947494886	-1,356942335	0.391202730434102	-3.46864229068867	0.000523095436612954	0.0485451939081881
ZNF730	1514.0697806486	-1,344838186	0.313837162227694	-4.28514640112768	1.82618890044594e-05	0.00644490356034845
ZNF117	19175.6468186993	-1,307338968	0.339167485665407	-3.85455276117189	0.00011594137916063	0.0220086601335447
NEDD9	1990.17199699392	-1,283453248	0.368284315402898	-3.48495223407801	0.000492224901728403	0.0474351276244829
MREG	149.741061125725	-1,194883579	0.304310093787563	-3.9265328457526	8.61791286025078e-05	0.018299918859263
RPL15P18	28.3530952662166	-1,147799493	0.306714808670328	-3.74223696037054	0.000182389405746991	0.0286160549173221
RP11-317B7.2	29.514466078877	-1,12221945	0.301235862559566	-3.72538462260204	0.000195017637630128	0.0295627695990895
SLC39A10	3721.35039501529	-1,098436313	0.289665522216485	-3.79208510712079	0.000149387696663269	0.0251221980892049
SNX6P1	47.661096314601	-1,092132914	0.312973515141283	-3.48953780929832	0.000483856605230449	0.0471750776547057
RPL13AP20	34.2686430367449	-1,080537161	0.285216458948325	-3.78848108844007	0.00015157112812139	0.0253194517155844
DCBLD2	5165.75338403111	-1,059496188	0.30427635073815	-3.48201950357852	0.000497647444858899	0.0474351276244829
TMPRSS11D	215.79178186261	-1,043956541	0.283579073189009	-3.68135959277926	0.000231993535854908	0.0326211454460878
AC138783.12	229.57229918598	-1,00373321	0.28708349390967	-3.49631111363569	0.00047173834114466	0.0467207415575563
RP11-43D2.2	68.0677783535827	-0,956529121	0.274715244868014	-3.48189312066638	0.000497882370804127	0.0474351276244829
PON2	5561.85389584855	-0,955534836	0.221252088487975	-4.31876074951138	1.56907764773488e-05	0.00601978914668503
SIX4	448.461233492254	-0,953321046	0.260579425578922	-3.65846629530704	0.000253729100741516	0.0334903064223998
RNF24	204.997226225798	-0,911412684	0.248292493968472	-3.67072185563668	0.000241866429732804	0.0329888893865152
GNRHR	292.453294150853	-0,896676237	0.244518906231127	-3.66710390955834	0.00024531312657572	0.0330473710355259
FNBP1L	3045.10201203527	-0,875151887	0.212635224808503	-4.11574275930925	3.85934551887329e-05	0.0100732938194175
LRRC41	2154.95707717197	-0,861219765	0.245823507178366	-3.50340687565337	0.000459347237819861	0.0458272610478414
RP1-95L4.4	52.3342708635547	-0,81242006	0.224940405479072	-3.61171243526205	0.000304181760558239	0.0360646625702077
ZNF713	175.689993359043	-0,804372899	0.21994744202033	-3.65711413448182	0.000255070823146512	0.0334903064223998
ZNF273	2705.39063615609	-0,790458075	0.215370480803981	-3.67022477705977	0.000242337272865884	0.0329888893865152
LYG2	110.773360952267	-0,747258881	0.203453945883816	-3.67286501813127	0.000239846191719244	0.0329888893865152
TMEM106C	1445.14558328032	-0,741336772	0.202839159037144	-3.65480105155377	0.000257381487212726	0.0334903064223998
ZNF138	1797.76757987621	-0,685606283	0.198264685408131	-3.45803531111628	0.00054413006134359	0.0497873769349827
SRPK2	2882.72107218599	-0,666858285	0.164720819787488	-4.04841528701139	5.15655913219921e-05	0.0127928616015362
GMCL1	1603.52317718167	-0,609173002	0.162481861059437	-3.74917543238121	0.000177416948229616	0.0284970286653172
CNPY2	209.094004355366	-0,604240316	0.16608773884273	-3.63807900568651	0.000274679172738125	0.0344131801564959
ZNF736	2854.76607161883	-0,596699153	0.168551604300117	-3.5401570662525	0.0003998889646604	0.0433293430462802
GPCPD1	2988.73142825145	-0,558226925	0.155348664075554	-3.59338091684449	0.000326414862493779	0.0376911392143163
ANKFY1	2413.11210768632	0,494875501	0.140261009162739	3.52824711527334	0.000418321436734197	0.0442981936123293
SOD2	1285.95204520411	0,501487433	0.141266992299018	3.54992645582158	0.000385338784644709	0.0419801475080108
TPCN2	1452.76501376558	0,587679542	0.158026557705633	3.71886568255596	0.000200119423527541	0.0295627695990895
DDB2	1234.08452077722	0,597057671	0.169485328388501	3.52276905863665	0.000427063314301669	0.044773746721577

IL17RC	514.432110172253	0,615342179	0.1675455822443	3.67268519436593	0.000240015091029904	0.0329888893865152
INPP5A	1172.24814730037	0,665285741	0.181096546061816	3.67365228902595	0.000239108060153633	0.0329888893865152
SF3A2	547.1472370713	0,693749239	0.198774154818322	3.49013803968507	0.000482771108167094	0.0471750776547057
SP140L	1091.55361778389	0,726657555	0.203554493015104	3.56984286812123	0.000357195421320096	0.039956453892936
IL17RA	1806.26207621893	0,7303518	0.19095214179899	3.82478977964467	0.00013088371871279	0.0234253809984741
CD37	396.716854584866	0,75182008	0.190501587689912	3.9465292082473	7.92922527855051e-05	0.0174282980530386
ENGASE	375.793165824899	0,758702116	0.216461597504276	3.50501948134435	0.000456573872608251	0.0457614861037797
CASP1	647.222437206704	0,774212583	0.213375485337325	3.62840455428362	0.000285178142387366	0.0347391843046664
ARRB1	685.80127740396	0,796372542	0.219154846937673	3.63383494926298	0.000279239530340844	0.0347391843046664
KCNAB2	197.424039278842	0,829709643	0.222672469308968	3.72614380989058	0.00019443150081099	0.0295627695990895
ERVK13-1	62.8275353546719	0,878760124	0.254354439877869	3.45486449649292	0.000550569453032164	0.0498036779228409
CD274	108.368456770335	0,957248218	0.217760604476826	4.39587417635024	1.10327832129145e-05	0.0052160084710566
GMFG	156.222179012802	0,975649997	0.276558626460985	3.52782341091689	0.000418991574654669	0.0442981936123293
PIK3CD	1114.4646615788	0,99875776	0.273908979179199	3.64631259133736	0.000266030334059326	0.0340098065332884
FGR	98.0665773131421	1,020814404	0.290693841924576	3.51164784496933	0.000445337680105907	0.0454712459160532
XAF1	2113.63191177302	1,024745799	0.275282765350492	3.72252072251035	0.000197243714832709	0.0295627695990895
HCST	50.2215135303229	1,037600753	0.294415412088328	3.52427458052083	0.000424643957784373	0.0447071581941304
GIMAP4	151.348161007357	1,095634935	0.30182322294513	3.63005511750928	0.000283360705353187	0.0347391843046664
SP140	356.076000273888	1,097833756	0.311146987065706	3.52834448570769	0.000418167575530899	0.0442981936123293
PTPRC	5086.27655119168	1,112576459	0.31526573266851	3.52901170068684	0.000417114687790013	0.0442981936123293
ABI3	27.2649577349234	1,13178504	0.307355326733334	3.68233422864218	0.00023110814427205	0.0326211454460878
CD53	602.505130780851	1,133905451	0.279719023887635	4.05373018807764	5.0407351682339e-05	0.0126305701110437
AMICA1	246.836057517405	1,147829721	0.3022604560664	3.79748557264774	0.000146171281354762	0.0251221980892049
DOCK8	3485.03723338515	1,149408503	0.246577915236081	4.66144140128139	3.14002474705735e-06	0.00213703661458793
FGD2	136.98704731213	1,154194265	0.260188765906845	4.43598808196065	9.16509186902947e-06	0.00478436889504732
AKNA	1292.26869452363	1,160000714	0.296248716496005	3.91563118889151	9.01679978662524e-05	0.0189860464078545
CD6	120.197629752767	1,166327973	0.277090005439456	4.20920260696758	2.56273510436236e-05	0.00793482018197579
HLA-E	6645.97036808082	1,190642237	0.342489161347498	3.47643771451925	0.000508122239181675	0.0476671451997152
DNAJC5B	14.2858962976473	1,20477331	0.348413086037111	3.45788765857402	0.000544428354559016	0.0497873769349827
IL18RAP	21.9971658249276	1,205932686	0.331984822709798	3.63249342650212	0.000280695728720965	0.0347391843046664
APOL3	306.049196342451	1,215509568	0.296217705079595	4.10343320737301	4.07064009778226e-05	0.0105152607144464
HMHA1	124.363023035558	1,221631016	0.315999708121415	3.86592450707843	0.000110669169727773	0.0214964138439442
C10orf54	231.392388846428	1,230519714	0.328860017589505	3.74177354701281	0.000182726135881053	0.0286160549173221
APOL1	565.538575098349	1,230686972	0.351372370873648	3.50251492199815	0.000460887966797942	0.0458272610478414
PRR24	45.5328403941212	1,236173257	0.32956415229777	3.75093361360601	0.000176177345404656	0.0284804886697062
CTSS	932.040426734501	1,247805119	0.235523814746438	5.29799978298937	1.17078105822355e-07	0.000245671398953828
SLAMF8	30.1428681975918	1,264543534	0.360779463443759	3.5050319167481	0.000456552546994023	0.0457614861037797
BIRC3	600.600841851028	1,266087718	0.293317117796885	4.31644674276293	1.58560914587226e-05	0.00601978914668503
IRF1	992.898124276231	1,274618058	0.310865314556403	4.10022604018853	4.12746790751037e-05	0.0105532615671926
CCR7	27.4128670356147	1,275143087	0.365919614266317	3.48476287554825	0.00049257335009326	0.0474351276244829
ATP2A3	353.154228935068	1,277776155	0.356802487913975	3.58118622478926	0.000342037742966475	0.0386055843491485
SLC2A5	72.2984070167071	1,279276975	0.355213643810439	3.60142972333652	0.000316471975113973	0.0367122142612539
RASAL3	59.5884397748011	1,302770244	0.350221446328456	3.71984713502578	0.00019934339290605	0.0295627695990895
RAC2	422.921478813855	1,328801391	0.350310282497661	3.79321263872023	0.000148710705651824	0.0251221980892049

IDO1	181.13995715257	1,336108998	0.382405414163072	3.49395941748168	0.000475913356699888	0.0469486652709807
GBP4	1240.42650886656	1,352356895	0.353204476827876	3.82882149937208	0.000128758349610559	0.0233789707695056
TNFRSF14	412.6307305674	1,354529853	0.385914241220856	3.50992450649139	0.000448233936275897	0.0454712459160532
CYTIP	239.639409429422	1,358847469	0.371898526815775	3.65381245557746	0.000258375024145397	0.0334903064223998
LY9	40.8255982972444	1,36163544	0.392503798445594	3.46910130625686	0.000522202502877968	0.0485451939081881
ITGAD	66.6293952371481	1,362112171	0.36173893712527	3.76545633158377	0.000166245178131533	0.0270639070899652
IL15RA	226.933407975286	1,363675531	0.378637666305645	3.60153162884025	0.000316347925902783	0.0367122142612539
CD48	98.760743036872	1,367250058	0.339828569281948	4.02335230746378	5.73755697078255e-05	0.013844027507637
FCN1	119.498222033673	1,402844872	0.378549148772122	3.70584606056537	0.000210686241849093	0.0306928207093762
SPN	36.0505927434081	1,412252618	0.316031248838506	4.4687119497783	7.86920150293604e-06	0.00428649091432757
PARP15	309.105414506132	1,426787716	0.386589456680947	3.69070519421185	0.000223633203964787	0.0321913391670002
LSP1	217.210903191977	1,432579469	0.322523590277867	4.44178197247911	8.92169356143443e-06	0.00475640160784814
LYZ	1668.94777048818	1,448579615	0.276666213705272	5.23583850631316	1.64237512898378e-07	0.000245671398953828
CCL22	6.82149599787013	1,449689193	0.417029903592016	3.47622360172815	0.000508528107781961	0.0476671451997152
IL12RB1	94.8467792314126	1,460931219	0.344790085751516	4.23716133158444	2.26363488742883e-05	0.00766485126679788
NCF1	343.977861555494	1,461928837	0.296948144396251	4.92317889489075	8.51495446942979e-07	0.000826903320229193
HLA-F	1899.13955349451	1,46593638	0.385766009981853	3.80006621166798	0.00014465743212761	0.0251221980892049
GRAP2	37.3465214431433	1,494848002	0.372838521399217	4.00937112478537	6.0880668741234e-05	0.0143913860061236
CCR2	87.532530721693	1,502406031	0.401166523193527	3.74509323105194	0.000180326798178113	0.0286160549173221
SCML4	20.9140749920636	1,506660005	0.413653063734529	3.64232768211244	0.000270183836564145	0.0341925851835221
AQP9	67.6745169461121	1,509696823	0.434377854137239	3.47553819541502	0.000509829385541912	0.0476671451997152
SNCG	217.073379312172	1,511823515	0.432942782113633	3.49197071267133	0.000479470816948533	0.0471141186677623
CTLA4	37.0170187354861	1,51473518	0.435433288933009	3.4786848375128	0.000503880807917287	0.0476671451997152
KCNB1	62.5784722627469	1,519988826	0.412069351369829	3.6886723572235	0.000225427291966941	0.0321913391670002
PADI2	81.2042831365228	1,530651856	0.403150627081109	3.79672448357411	0.000146620590581686	0.0251221980892049
PTPRH	77.6911638050796	1,547164393	0.402651881794678	3.84243676252331	0.000121818806948568	0.0226104729311872
RILP	54.2973451418138	1,548558998	0.359399124102578	4.30874449564207	1.64183907970362e-05	0.00614023310748265
STX11	32.7245621500317	1,551477175	0.383674760660305	4.04373009034423	5.26074786183339e-05	0.0129233881543097
BAI1	235.104516862951	1,560602347	0.451558481451833	3.45603595242306	0.000548182198952031	0.0498036779228409
SYCP2	942.699147272607	1,565809679	0.438816189319754	3.56825868626143	0.000359361614920177	0.039966553372524
CD8A	324.827744265324	1,580838735	0.383722404526453	4.11974572375099	3.79290738858312e-05	0.010004092677445
GZMA	67.6128262744247	1,581300103	0.39569414833689	3.99626860783005	6.43487180833955e-05	0.014931811720933
CCR5	67.4504476647688	1,588023857	0.301251032862812	5.27143041282391	1.35364610427381e-07	0.000245671398953828
NTM	53.0446624985048	1,591393359	0.446332128147587	3.5654913879729	0.000363175088210886	0.0400884501555073
CD247	148.27493288396	1,595029952	0.338787226220017	4.70805812230206	2.50087800180111e-06	0.00190189859537512
RLTPR	67.7718597894432	1,621352498	0.381631346913058	4.24847830405899	2.15227486236119e-05	0.00738760975701154
PRF1	94.2473358449838	1,625370968	0.348396531009366	4.66529033174273	3.08181593713231e-06	0.00213703661458793
SLAMF6	76.1268320987578	1,627795994	0.422995933269588	3.84825447651072	0.00011896244348103	0.0222450891515236
BCL11B	20.8719938461119	1,64266845	0.4284982706901	3.83354744215559	0.000126308426838321	0.023101534680933
GZMB	49.6180518708285	1,642711938	0.411289945554471	3.99404837333038	6.49546026093184e-05	0.014931811720933
ITGAL	359.115085518535	1,655380332	0.315812855626136	5.24164961186286	1.59147361411748e-07	0.000245671398953828
TRGV10	18.6961275477023	1,658858408	0.480132089298637	3.45500424738389	0.000550284153486568	0.0498036779228409
KLRC1	28.3504447390345	1,66731401	0.402493705413179	4.14245983785363	3.43600552308443e-05	0.00966163748938818
TMEM156	58.6173959422492	1,670391155	0.449612982068736	3.71517554247859	0.000203062673782933	0.0297552129647892

AC006116.20	2.53037977260533	1,676199332	0.476252618280119	3.51955930107874	0.000432264390866408	0.0451302035080816
CD38	160.421967355244	1,680211595	0.345057062352506	4.86937315248343	1.1195282159798e-06	0.00103896364843726
EOMES	44.5322280313403	1,692812712	0.437369675627725	3.87043914161003	0.000108639476404613	0.0214344831517354
FASLG	22.6699563622117	1,697419206	0.385088177619744	4.40787150876618	1.04391441613612e-05	0.00503026221636978
GPR174	72.0475226859645	1,700584824	0.416789280504273	4.08020288224396	4.49964105504888e-05	0.0113886369612485
CD2	149.848721589116	1,709143366	0.399351397879308	4.27979812950633	1.87062907416217e-05	0.00651004898767798
TRAJ14	3.97188201488084	1,711191185	0.474055863242939	3.60968256654269	0.000306571941072524	0.0360646625702077
IGHV1OR15-1	33.7811140700065	1,712967268	0.48076922281847	3.56297197555699	0.000366679837495343	0.0401218195987808
GFI1	22.1410962951845	1,71382264	0.396518065137333	4.322180478064	1.54494728925453e-05	0.00601978914668503
TRAC	104.148983370784	1,71693072	0.409344045905527	4.1943463868173	2.73659529049337e-05	0.00816319859451101
TRAV4	4.36681580592729	1,7241329	0.490355863457086	3.51608500825573	0.00043796073304379	0.0451604201147253
CD3G	105.210982059769	1,733323947	0.41353833977545	4.19144679104935	2.77181198883803e-05	0.00817097564756641
ITK	219.284773702598	1,735983994	0.395336146080608	4.39115929767403	1.12747897366914e-05	0.00523171123022733
ZNF831	32.1697848093016	1,767169776	0.446321806190391	3.95940720747775	7.51360305978237e-05	0.0168096742740149
TRAJ11	6.31861912982421	1,771307222	0.485475628245544	3.64860174034441	0.000263671482530324	0.0338811094244222
CD3D	219.93278029524	1,775562318	0.441352193566719	4.02300553597203	5.74601453004849e-05	0.013844027507637
SSTR1	5.9023463631524	1,78189865	0.493539993174676	3.61044428958542	0.000305672954346987	0.0360646625702077
IL2RB	72.2354298035493	1,782394706	0.414469828443343	4.30042088292052	1.70474026656851e-05	0.0062245176399535
P2RY8	28.1330472941505	1,793956733	0.481880301841107	3.72282644995279	0.000197004942943983	0.0295627695990895
IL2RG	164.994330299904	1,799498071	0.342359773210071	5.25616094958154	1.47093571978806e-07	0.000245671398953828
POU2AF1	62.5178046892081	1,800440819	0.490725658941762	3.66893555712452	0.00024356245905357	0.0329888893865152
ZNF683	19.8859084781645	1,807473302	0.493531414992409	3.66232674685009	0.000249934783683635	0.0333117865678768
LCK	64.0129159652889	1,8099434	0.373223892209744	4.84948428461867	1.23782875530559e-06	0.00106952672833421
KIF19	35.3996247442933	1,811700274	0.491130670002735	3.68883554735096	0.000225282770648767	0.0321913391670002
CD3E	306.159555575891	1,829143207	0.393196606401409	4.65198116369684	3.28761115948967e-06	0.00216783349535086
CCL5	203.720581018729	1,837745243	0.382437375248169	4.80534948163309	1.54481421072402e-06	0.00124865837671328
KIAA0125	70.510565600031	1,840553515	0.510729897591379	3.6037708459517	0.000313633596947663	0.0367122142612539
IGHM	2792.92702208638	1,845482687	0.516211905519941	3.57504867171878	0.000350162748058626	0.0393454169421749
THEMIS	52.3431922024398	1,848511814	0.440390924183131	4.19743394444443	2.69956360415861e-05	0.00814975484691594
PRKCG	182.94143389345	1,859059261	0.480228152439847	3.87120007681172	0.000108300852439076	0.0214344831517354
TRBC2	244.330595294374	1,881403523	0.356136048542523	5.28282247937781	1.27208643376215e-07	0.000245671398953828
RP11-313M3.1	4.30051306615048	1,886341766	0.526378699820282	3.58362100554255	0.000338863669617659	0.0385844203478838
CHRDL2	34.430967250587	1,891252237	0.478565793135718	3.95191688136243	7.75276707790546e-05	0.0171912464310688
UBASH3A	30.4931643365522	1,892285074	0.440146879906693	4.29921274054903	1.71405881453004e-05	0.0062245176399535
ASS1P1	10.376824603473	1,895634141	0.498336855591642	3.80392122278357	0.000142423489462937	0.0249559816466629
LAX1	86.8883768186615	1,898737081	0.43771464081091	4.33784229233706	1.43888338876954e-05	0.00581517759232233
IGKV6D-21	12.9811911034324	1,910724049	0.549640088850159	3.47631857181291	0.000508348046807431	0.0476671451997152
IGKV2-30	243.456959308995	1,925770939	0.547582337494433	3.51686094810142	0.000436682481927736	0.0451604201147253
TNFRSF9	19.3786401437184	1,926298231	0.413331300654332	4.66042186450111	3.15561937740964e-06	0.00213703661458793
TRAV22	2.90727525613719	1,927180644	0.548925258324098	3.51082522642893	0.000446717993480308	0.0454712459160532
TNN	17.6726421764041	1,929380074	0.528067013748171	3.65366520488226	0.000258523318141196	0.0334903064223998
IGLV3-19	132.208312787926	1,930179688	0.518121947758253	3.72533859280711	0.000195053228647182	0.0295627695990895
IGHD3-10	3.06906455275582	1,933251835	0.539457451487391	3.58369660112076	0.000338765562834262	0.0385844203478838
IGHV1-3	221.709243364616	1,948499483	0.550531623067945	3.53930528503457	0.000401181609400048	0.0433293430462802

IGHV3-11	2142.57667934794	1,953529149	0.545296424980412	3.58250863166973	0.000340310368235715	0.0385844203478838
AADACL4	6.19200104051286	1,959498064	0.520406472272998	3.76532224004341	0.000166334425184765	0.0270639070899652
ZBP1	96.0631155439021	1,962173151	0.374953153029359	5.23311548514365	1.66676528802932e-07	0.000245671398953828
ENTHD1	3.83282596167836	1,967923426	0.525839757643843	3.74243939864562	0.000182242491149381	0.0286160549173221
IL26	2.83099995996877	1,9719789	0.544226376449031	3.62345337497458	0.000290695624946223	0.0350190397801803
IGLV4-69	42.4745815530548	1,974453406	0.50617831330469	3.90070722892939	9.59120854218609e-05	0.0196989272493079
IGKV2-28	6.88470908746825	1,982570581	0.545920151518519	3.63161274674425	0.000281655556210314	0.0347391843046664
IGHV4-39	885.986583793455	1,989997188	0.55057456251209	3.61440088813452	0.000301042949061552	0.0359201579744539
IGLV2-11	548.976963392121	1,993583554	0.533093069414213	3.73965385782879	0.000184273830256283	0.028679188600818
IGHV4-4	234.776762927687	1,995328377	0.5499767563133	3.62802310148546	0.000285599711328622	0.0347391843046664
IGLL5	7.24156527142428	2,018439125	0.542224299545033	3.72251691145387	0.000197246692967501	0.0295627695990895
SLAMF1	47.2050477207372	2,036073626	0.43932075428733	4.63459466854902	3.57637540026579e-06	0.0022977753437041
IGHV3-23	1918.78450994508	2,037083683	0.532554568247267	3.82511728325921	0.000130709845161177	0.0234253809984741
TRAJ22	3.8374646255852	2,03747811	0.455670914237949	4.47138065189623	7.771622715471e-06	0.00428649091432757
IGKV4-1	1153.10387671614	2,040878874	0.548874469185536	3.71829806030419	0.000200569534734614	0.0295627695990895
TRAV21	2.30641932440759	2,054214115	0.522322245449169	3.93284822245742	8.39452452455819e-05	0.0181074411422425
IGKV1D-12	405.383192381996	2,07921577	0.54813886658599	3.79322813431054	0.000148701421963067	0.0251221980892049
IGLV3-25	254.117553412201	2,084043125	0.543406503863685	3.83514571463569	0.000125489874639842	0.023101534680933
IGHV5-51	432.453848877785	2,0851227	0.548041228785884	3.80468218494467	0.000141986376281312	0.0249559816466629
IGHJ5	36.9841173803529	2,105049387	0.535301251534215	3.93245743557656	8.40818712408612e-05	0.0181074411422425
IGHV3-73	551.317368543683	2,108279885	0.539402010949802	3.90855028816315	9.28516214314687e-05	0.01926963206053
IGHV6-1	14.3639601515547	2,108725252	0.54624744614757	3.86038464191129	0.000113208677471984	0.0216539681787442
IGHV3-48	1753.13937670748	2,117316357	0.54748530153103	3.86734831253429	0.000110025223640955	0.0214964138439442
IGKV3-15	1694.39339918695	2,121730497	0.549203025518253	3.86328989212964	0.000111870116947058	0.0215625347718649
AC104297.1	35.6239370099855	2,123809689	0.547050493980844	3.88229187677816	0.000103476568825376	0.0207424990804596
IGLV5-45	28.386198266632	2,126671484	0.532354345686925	3.99484197171091	6.47374184365587e-05	0.014931811720933
IGHV1OR15-3	22.3611244826767	2,134408749	0.489030339211243	4.36457327413747	1.27371150961363e-05	0.00550265332696356
IGHG1	149254.015221778	2,148275041	0.534735588333439	4.01745290149554	5.88305888065435e-05	0.0140392196545292
GZMK	20.1894051804341	2,155597365	0.390353658074575	5.52216514426105	3.34847536012059e-08	0.000117249618279957
IRF4	111.709113249662	2,15634692	0.467381685869707	4.6136744011421	3.95612010020748e-06	0.00244374299407604
IGHGP	1775.78568079087	2,212326852	0.534884408365524	4.13608401600044	3.53282889373259e-05	0.00966163748938818
IGLV1-40	753.058660782494	2,239231832	0.541501120972534	4.135230279841	3.5459887565166e-05	0.00966163748938818
PLA2G2D	37.780285674532	2,249041117	0.537633312754886	4.18322500480671	2.874026863235e-05	0.00827752771403212
IGKV1-27	1787.07096654226	2,2722026	0.539185965924834	4.21413527675797	2.50736976112926e-05	0.00793482018197579
CD79A	117.102637057737	2,275531697	0.514660497893483	4.42142287241532	9.80530645123602e-06	0.00481748164212982
IGKV3D-20	59.9512404935567	2,28268754	0.541742360193773	4.21360356489219	2.51328264111407e-05	0.00793482018197579
IL21R	17.7864422972214	2,288190176	0.452132037224307	5.06088927130113	4.17305526645534e-07	0.000522821229057858
IGLV3-9	126.202052682136	2,311485855	0.548307762220368	4.21567231773126	2.49035148428857e-05	0.00793482018197579
IGKV2-24	349.270118563438	2,314939266	0.550301638943567	4.20667339968962	2.59157251054343e-05	0.00793482018197579
MZB1	48.8733830696009	2,318770974	0.449964593419288	5.15322984978753	2.56037822010132e-07	0.000337659984531993
GNLY	68.8656983010716	2,324023359	0.471680328614624	4.92711529035909	8.34525181387636e-07	0.000826903320229193
IGKV3-20	7268.05080312297	2,364187305	0.541277771447885	4.36778938562545	1.25510437270483e-05	0.00550265332696356
IGHV1-69	1069.20973306343	2,368627818	0.542042774668995	4.36981716053432	1.24350611047071e-05	0.00550265332696356
SLAMF7	260.146328556421	2,38981502	0.419790470957119	5.69287581610642	1.24917244876303e-08	0.00010433504682885

IGHV1-24	444.026861013739	2,419674403	0.546258498154275	4.42954097936625	9.44338684926707e-06	0.00481748164212982
IGHG3	4060.2164942002	2,424211763	0.499111970930593	4.85704992864674	1.19147558599637e-06	0.00106624299136825
IGKV1-16	1034.87934023164	2,451667921	0.539160643155289	4.547193775427	5.43659391624089e-06	0.00316801706416856
IGJ	1258.05190344747	2,483730476	0.516610193210927	4.80774577994514	1.52641698997733e-06	0.00124865837671328
IGLV2-23	2373.21473270116	2,486131315	0.548290153224497	4.53433515066468	5.77852122906734e-06	0.00329073650992592
IGHV3-30	956.289340835451	2,505992199	0.547064977090577	4.58079442767827	4.63212991530525e-06	0.00276350664970961
IGLV1-47	307.381221332333	2,509025164	0.536678943134278	4.67509522363223	2.93817255610057e-06	0.00213703661458793
IGHV3-53	743.285828643113	2,509434325	0.544174208297173	4.61145399200949	3.99862165291606e-06	0.00244374299407604
IGKV1-6	985.649511164987	2,574371094	0.520081651685438	4.94993639187367	7.42377404737483e-07	0.000808771766543788
IGKV1-12	835.203565782078	2,580698599	0.517450737586802	4.98733195492061	6.12188276854663e-07	0.000730457221578442
IGHV3-49	311.272093331258	2,636717354	0.510341079203897	5.1665787078494	2.38417829242659e-07	0.000331890863740739
IGHV1-18	1417.71152202653	2,664749904	0.541430424381325	4.92168482623252	8.5802316023303e-07	0.000826903320229193
IGHV1-2	1304.75298927307	2,673826074	0.539032529453022	4.96041691065097	7.03420448198831e-07	0.000801163916841732
IGHV2-70	58.011356677519	2,876944375	0.547773295568011	5.25207124635577	1.50398260314049e-07	0.000245671398953828
CXCL9	161.97659389416	2,878537208	0.404496591152729	7.1163447875232	1.10826850861255e-12	2.77698840203047e-08
IGHV3-33	2141.20526698403	2,986032658	0.5495857321064	5.43324268429358	5.53390627610687e-08	0.000154070099511567
IGHV4OR15-8	70.5047033635657	3,001210932	0.539261454168721	5.56540970805889	2.61537180023816e-08	0.000109222285330946
IGHV4-31	681.006063874587	3,008423135	0.539008767381962	5.58139925872357	2.38591236160941e-08	0.000109222285330946
IGHV3-21	2175.18481255203	3,033383487	0.538247645796124	5.63566512686516	1.74383955574137e-08	0.000109222285330946

Supplemental Table 4. **List of genes significantly overrepresented in DC-LAMP^{Hi} versus DC-LAMP^{Lo} HGSC samples as per RNA-Seq.**

Supplemental Table 5

Variable	Study group 3 (n=20)
**Age:**	
Mean age (y) ± SEM	60.9 ± 2
Range	44-78
**pTNM stage:**	
Stage I	0 (0%)
Stage II	6 (30%)
Stage III	14 (70%)
**Debulking**	
R0	8 (40%)
R1	1 (5%)
R2	11 (55%)

***Supplemental Table 5*. Main clinical and biological characteristics of 20 HGSC patients in which the freshly resected tumors were analyzed using flow cytometry (University Hospital Motol).**

Supplemental Table 6

**Variable**	**Cohort for NGS** (n=18)
**Age:**	
Mean age (y) ± SEM	62.9 ± 2
Range	44-78
**pTNM stage:**	
Stage I	1 (5.6%)
Stage II	1 (5.6%)
Stage III and IV	16 (88.8%)
**Debulking**	
R0	7 (38.9%)
R1	3 (16.7%)
R2	8 (44.4%)
**Vital status of patients**	
Alive	3 (16.7%)
Dead	15 (83.3%)

***Supplemental Table 6*. Main clinical and biological characteristics of 18 HGSC patients whose tumor samples were used for NGS data analysis (University Hospital Motol).**
